# A multi strategy optimization framework using AI digital twins for smart grid carbon emission reduction

**DOI:** 10.1038/s41598-026-38720-3

**Published:** 2026-02-12

**Authors:** S. Sakthivel, M. Arivukarasi, G. Charulatha, J. Nithisha, B. Abirami, A. K. Jaithunbi, V. Suresh Kumar

**Affiliations:** 1 Department of Electronics and Communication Engineering, Saveetha School of Engineering, Saveetha Institute of Medical and Technical Sciences (SIMATS), Chennai, Tamil Nadu 602105 India; 2 Department of Computer Science and Engineering, Saveetha School of Engineering, Saveetha Institute of Medical and Technical Sciences (SIMATS), Chennai, Tamil Nadu 602105 India

**Keywords:** Digital twin, Carbon neutral smart grids, Multi energy storage, AI optimization, Model predictive control, Renewable energy, Energy science and technology, Engineering, Mathematics and computing

## Abstract

This research presents an AI-enabled digital twin framework to achieve carbon neutrality in smart grids through optimal management of heterogeneous energy storage systems. The proposed structure integrates battery, thermal, and hydrogen storage technologies with AI-driven forecasting models to address the challenge of renewable integration, while maintaining grid stability and economic viability. This paper presents a comparative analysis of three distinct optimization methodologies, like a rule-based (RB) heuristic approach, Model Predictive Control (MPC) with look-ahead capability, and a multi-objective Genetic Algorithm (GA). Simulation results that demonstrate the AI-optimized multi-energy storage (MES) integration significantly enhance the renewable utilization and reduce carbon emissions by approximately 30% compared to conventional approaches. Specifically, the MPC achieves a 29.9% reduction in carbon footprint (1741.1 kgCO₂ vs. 2485.2 kgCO₂ baseline) with corresponding operational cost savings of 30%, while GA shows a comparable 28.2% improvement. The comparative analysis discloses a critical trade-off between computational complexity, optimization performance, and practical implementability, with MPC emerging as a balanced method for a real-world application. This work has contributed to sustainable energy systems by providing a comprehensive framework for MES optimization, imparting treasured insights for grid operators and policymakers. The outcomes highlight the important role of AI-enabled digital twin in designing next-generation smart grid infrastructure, which is capable for supporting excessive renewable penetration at the same time as ensuring reliability and sustainable economic growth.

**Supplementary Information:**

The online version contains supplementary material available at 10.1038/s41598-026-38720-3.

## Introduction

### Research motivation

The global transition closer to a sustainable energy future is unequivocally connected to the decarbonization of the power sector^1^. This imperative has catalysed the rapid integration of the renewable energy sources (RES), which include solar and wind power, into modern-day electrical grids^2^. While RES are pivotal for reducing greenhouse gas emissions, their inherent intermittency poses significant challenges to grid stability and power quality^3^, with stochastic nature complicating reliable operation^4^. This variability creates a critical mismatch between the energy generation and the energy consumption, which often leads to renewable curtailment during periods of excess production and reliance on carbon-intensive peaking plants during deficits^5^.

Energy storage systems (ESS) are widely recognized as a cornerstone technology for mitigating these challenges^6^, enabling temporal arbitrage by storing surplus renewable generation^7^. Traditionally, research and deployment have focused on a single storage type, most notably lithium-ion batteries, prized for their high power density and rapid response^8^. However, a single-technology approach has some limitations. Batteries are ideal for short-to-medium duration storage, but they are less economically viable for long-term, large-scale energy shifting due to the cost and degradation concerns^9^. This has led to the emergence of multi-energy storage (MES) systems, which advocate for synergistic integration of heterogeneous storage technologies^10^ to leverage complementary characteristics^11^. This paradigm leverages the complementary characteristics of various systems: batteries for high-power, short-duration applications; thermal storage for efficient industrial heat management; and hydrogen storage for seasonal, long-duration energy storage^12^. Optimizing the dispatch of the sort of numerous portfolio is a complex, multi-objective control problem that must balance the carbon reduction, economic cost, and device lifetimes^13^.

The operational complexity of MES-integrated smart grids creates a necessity for advanced digital tools^14^. A digital twin, a virtual, dynamic replica of a physical system enabling simulation, analysis, and control, is a concept gaining significant traction in power systems engineering^15^. When it’s empowered by artificial intelligence (AI), these digital twins manage to transcend traditional simulation models^16^ they do this by incorporating predictive analytics, machine learning-based forecasting, and adaptive optimization, which ultimately creates a powerful platform for real-time decision support^17^.

Now, while the existing literature has certainly made substantial progress in exploring MES^18^ and also in applying digital twin to grid management^19^, a significant research gap remains. Previous studies often tend to focus on just a single optimization algorithm in isolation, whether that’s a simple RB heuristic^20^, an MPC scheme^21^, or maybe a metaheuristic approach like a GA^22^. What we find is that there is a clear lack of a systematic, comparative analysis that looks at these disparate strategies within a unified AI-enabled digital twin. The trade-offs between computational complexity, optimality, practicality, and performance for the specific problem of carbon–neutral operation with MES are simply not well-quantified at this point^23^. For grid operators on the ground, understanding whether the marginal gain of a complex AI algorithm truly justifies its computational overhead compared to a simpler MPC or heuristic approach remains a critical, and still unanswered, question.

To bridge this gap, this research introduces a new AI-driven digital twin designed to optimize the carbon–neutral smart grids that incorporate with MES. The work makes four primary contributions: First, it develops an integrated digital twin architecture. This framework uniquely combines the AI-based models for forecasting the load and renewable generation with high-fidelity simulations of various storage systems, which include battery, thermal, and hydrogen. Second, implement and directly compare the three different optimization strategies within this unified digital environment. These include a simple RB heuristic for ease of use, an MPC system for forward-looking optimization, and a multi-objective GA to map out Pareto-optimal solutions. Third, conduct a comprehensive analysis based on the extensive simulations. This evaluation rigorously assesses each strategy’s performance across several critical metrics: carbon footprint reduction, operational cost, computational demands, and the success of renewable energy integration. Finally, this study translates the findings into practical insights. It clarifies the inherent trade-offs between computational complexity and performance gains, providing grid operators with actionable guidance for selecting the most suitable strategy based on their specific priorities and resource constraints.

### Literature review

The global shift closer to decarbonization is fueling a rapid increase in the variable renewable energy (VRE) integration within the power systems across the world. However, this transition will bring significant challenges. As highlighted by^24^, the inherent intermittency and unpredictability of solar and wind resources have created substantial operational complexities for grid operators, including issues like frequency fluctuations, voltage instability, and a greater need for balancing reserves. These difficulties are exacerbated by the risk of renewable curtailment during periods of oversupply, a problem that, as^25^ stressed, can severely undermine the economic and environmental advantages of clean energy. In light of these challenges, a clear consensus has formed among researchers on the urgent need for advanced flexibility solutions to ensure grid reliability throughout the ongoing energy transition^26^.

Energy storage systems (ESS) have gained widespread recognition as a pivotal solution to these VRE integration challenges. Mancarella^27^ Provided a comprehensive review of various ESS technologies, classifying them according to their applications, from enhancing power quality to facilitating energy arbitrage. In particular, the batteries, especially lithium-ion, have proven effective for short-duration, high-power in frequency regulation applications^28^. Despite having these advances, Previous research has cautioned that^29^ reliance on a single storage technology introduces both economic and operational limitations, particularly for long-duration storage requirements. This realization has spurred a growing interest in MES systems, which leverage the complementary characteristics of diverse storage types. Early work by Geidl et al.^30^ introduced a hybrid system combining super capacitors with batteries, while subsequent studies expanded this concept^31,32^ expanded this concept by proposing integrated frameworks that incorporate electrochemical, thermal, and chemical storage, such as hydrogen, to build more resilient and cost-effective grid asset portfolios.

The challenge of optimally dispatching energy resources within the complex grid environments has long been a focus of research. Traditional methods often employ an RB heuristic or deterministic algorithms. For example, rule-based SOC strategies have been implemented^33^ a straightforward state-of-charge (SOC) based strategy for battery management, valued for its computational efficiency and ease of real-time implementation. To incorporate forecasting and adaptability, more advanced techniques like MPC have been widely adopted. MPC has been applied to microgrid energy management^34^ for microgrid energy management, using short-term predictions to optimize economic dispatch while maintaining system constraints, and^35^ extended this approach to developing a stochastic MPC framework to address the uncertainties in renewable generation. For particularly complex, non-convex, and multi-objective optimization problems, metaheuristic algorithms have shown considerable promise. GA have been utilized^36^ to simultaneously minimize the cost and emissions in a microgrid, while^37^ applied particle swarm optimization (PSO) to solve problems related to the optimal sizing and scheduling of ESS.

More recently, the digital twin concept is originally developed for manufacturing and aerospace that has begun to influence energy systems research. The digital twin concept, originally from manufacturing^38^ is a virtual replica of the physical asset, which is enabled by real-time data and dynamic models for simulation and analysis. Structured architectures for grid digital twins have been proposed^39^ structured architecture for a distribution of grid digital twin is emphasized, with its role in real-time monitoring and diagnostics. Building on this, Mbasso et al.^40^ integrated forecasting models into a digital twin for a wind farm, demonstrating its utility in supporting predictive maintenance^41^. The integration of artificial intelligence (AI) with digital twin represents a further evolution. Selvam et al.^42^ explored how machine learning can improve the predictive capability of a digital twin^43^, and^44^ developed an AI-driven agent for real-time anomaly detection within a grid-level digital twin environment^45^. A critical analysis of the existing literature reveals several interconnected gaps, as illustrated in Table 1. Recent advancements further demonstrate the field’s evolution toward integrated, resilient energy systems. Planning frameworks that combine renewable resources with battery storage enhance grid resilience^46^, while integrated approaches optimize both generation and carbon emission management^47^. Graph learning provides comprehensive solutions for power system robustness and antifragility^48^, and assessment methods for extreme event resilience continue to advance^49^. Multi-objective optimization now considers comprehensive energy systems with renewables, storage, and demand-side management^50^, while deep learning advances include spatio-temporal attention models for demand prediction^51^ and battery health forecasting for electric vehicle fleets^52^. These innovations collectively support the broader transition toward carbon-free energy societies^53^.

**Table 1:** Research Gaps and Proposed Work’s Contribution.

Research gap	Limitations in existing literature	Contribution of this work
Siloed Optimization Studies	Algorithms are evaluated on different systems and metrics, making direct comparison impossible	Provides a unified digital twin to implement and equitably compare RB, MPC, and GA strategies on an identical MES-smart grid system
MES Operational Control	Focus is often on technological potential or sizing, not on real-time operational control of heterogeneous assets	Develops and validates optimization strategies specifically for the real-time dispatch of a battery thermal hydrogen storage portfolio to maximize carbon reduction
AI-Digital Twin Convergence	Digital twin are often used for visualization and monitoring, not as a core component of an AI-driven optimization loop	Develops an AI-enabled digital twin that integrates forecasting models and hosts multiple optimization algorithms, demonstrating its value as a testbed for carbon–neutral strategies

### Research gap and novelty contributions

Previous studies have explored digital twins, multi-energy storage, and AI optimization individually, but this work addresses several key gaps through the following contributions:

#### Novel comparative framework

Provides the first comprehensive comparison of Rule-Based, MPC, and GA strategies within a unified digital twin framework using identical system configurations and performance metrics, unlike earlier studies that evaluate algorithms separately or on different systems.

#### Quantitative strategy trade-off analysis

Uniquely quantifies the trade-offs between computational complexity, optimization performance, and practical implementability for carbon–neutral operation, offering insights into whether advanced AI algorithms justify their computational cost for grid operators.

#### Integrated AI–digital twin architecture

Introduces a fully integrated digital twin that combines AI-based forecasting with multi-strategy optimization in a closed-loop control system specifically designed for carbon reduction in multi-energy storage environments.

#### Real-world validation with practical insights

Includes real-data validation and addresses practical implementation constraints, providing actionable guidance for deploying AI-optimized multi-energy storage systems at utility or community scale.

## Proposed digital twin and mathematical modeling

The proposed AI-enabled digital twin is designed as a closed-loop, with cyber-physical system that creates a dynamic virtual replica of a physical smart grid, which integrated with MES. Its primary function is to enable the simulation, analysis, and optimization of grid operations to achieve the carbon neutrality.

**Fig. 1:**
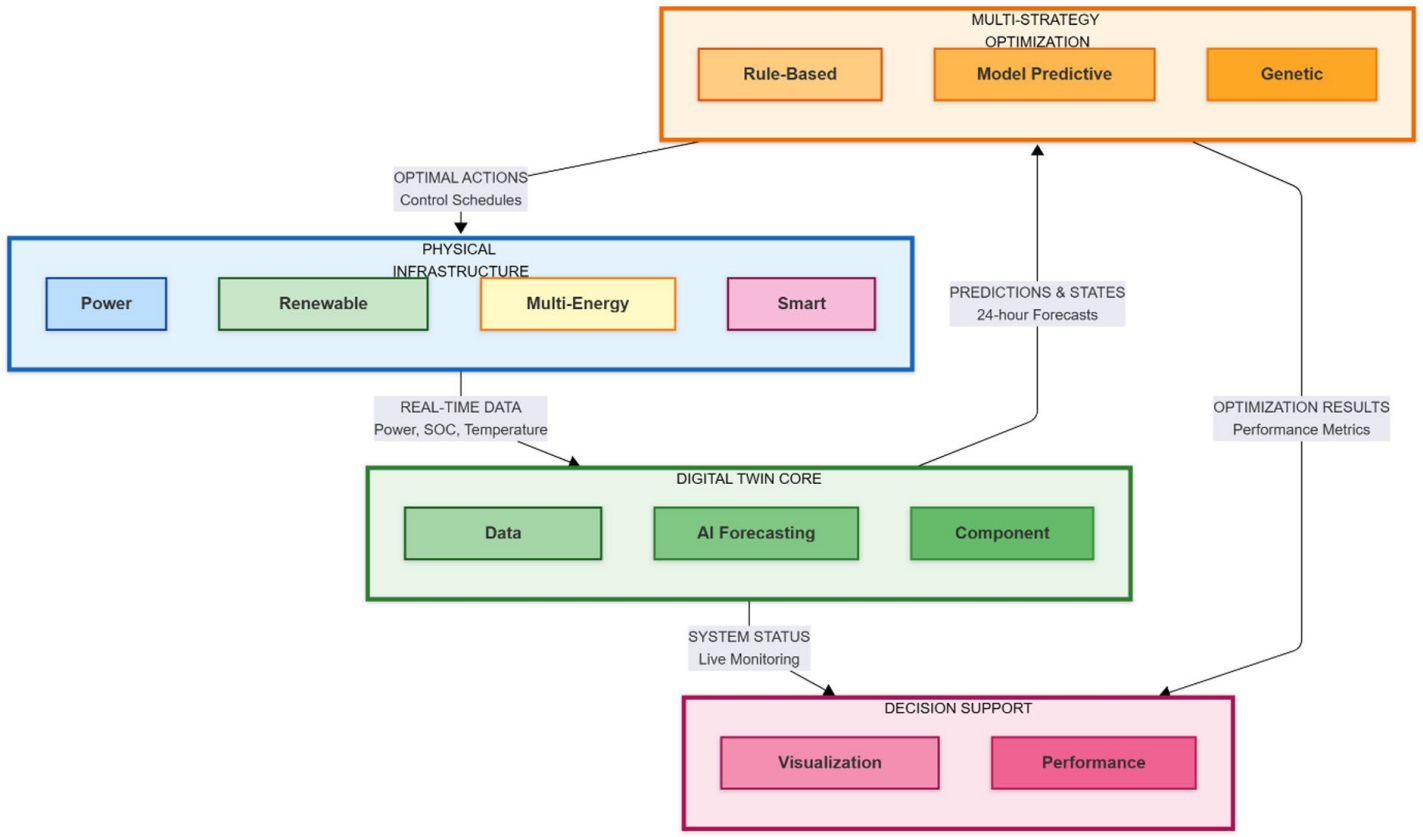
Architecture of the proposed AI-enabled digital twin for carbon–neutral smart grids.

The overall architecture, depicted in Fig. 1, consists of three integrated layers such as the Physical Layer, the Digital Twin Layer, and the Optimization & Control Layer.

### System architecture

#### Physical layer

This layer encompasses real-world grid infrastructure, inclusive of power generation sources (including renewables like solar PV and wind turbines), loads, and the MES system (battery, thermal, and hydrogen storage units). Sensors and smart meters will offer real-time data streams on key parameters such as power flows, SOC, temperature, and pressure.

#### Digital twin layer

This is the core of the framework. It ingests the data from the Physical Layer to maintain a synchronized and high-fidelity virtual model. This layer contains two key sub-modules: AI-Based Forecasting Engine, which utilizes the historical and real-time data to predict the renewable energy generation (solar, wind) and the load demand over a 24-h horizon. For this study, we employ an optimized neural network model trained on weather and load data. Component Modeling Suite hosts the mathematical models that emulate the behavior of each physical storage component. The Digital Twin Layer contains the AI-Based Forecasting Engine, which utilizes LSTM neural networks to predict renewable energy generation and load demand over a 24-h horizon with RMSE of 58.9 kW for load and 39.5 kW for solar forecasting. The Digital Twin Layer ingests data from the Physical Layer, utilizing both synthetic profiles (for controlled comparative analysis) and real validation data from Pecan Street Dataport to maintain synchronization with realistic operating conditions.

#### Optimization & control layer

This layer will host the optimization of strategies (RB, MPC, GA) that use the forecasts and models from the Digital Twin Layer to compute the optimal schedule for the MES system. Thus, resulting operational set points are then simulated within the twin for validation before being dispatched to the physical assets, ensuring secure and optimal operation.

### Mathematical modeling of components

The accuracy of the digital twin is contingent on the fidelity of its component models. The following mathematical models were developed for each storage technology.

#### Battery energy storage (BES) model

The BES is modeled considering its energy capacity and operational constraints. The SOC is the key state variable, evolving dynamically as:

**SOC dynamics:**

1 $${\mathrm{SOC}}\left(t+1\right)={\mathrm{SOC}}\left(t\right)+\frac{{\eta }_{\mathrm{ch}}\cdot {P}_{\mathrm{ch}}\left(t\right)\cdot\Delta t-\frac{{P}_{\mathrm{disch}}\left(t\right)\cdot\Delta t}{{\eta }_{\mathrm{disch}}}}{{E}_{\mathrm{cap}}}$$

where: ${\mathrm{SOC}}\left(t\right)$ is the SOC at time *t* (per unit, range 0–1). ${P}_{\mathrm{ch}}(t),{P}_{\mathrm{disch}}\left(t\right)$ are the charge and discharge powers at time *t* (kW). ${\eta }_{\mathrm{ch}},{\eta }_{\mathrm{disch}}=0.90$ are the charging and discharging efficiencies[Constant]. ${E}_{\mathrm{cap}}=1000$ is the total energy capacity (kWh). $\Delta t=1$ is the time step duration (hours).

The SOC is the key state variable, evolving dynamically as shown in Eq. (1)

**Operational constraints:**

2 $$0\le {\mathrm{SOC}}\left(t\right)\le 1$$

3 $$0\le {P}_{\mathrm{ch}}\left(t\right)\le {P}_{\mathrm{ch,max}}=200 [\mathrm{kW}]$$

4 $$0\le {P}_{\mathrm{disch}}\left(t\right)\le {P}_{\mathrm{disch,max}}=200 [\mathrm{kW}]$$

5 $${P}_{\mathrm{ch}}(t)\cdot {P}_{\mathrm{disch}}(t)=0\text{(Charge/Discharge Exclusivity)}$$

Operational constraints include SOC limits (Eq. 2), power limits (Eqs. 3–4), and charge/discharge exclusivity (Eq. 5).

**Battery degradation and cycle aging:**

We incorporate a throughput-based degradation model to account for battery aging costs:

6 $$\begin{array}{c}{C}_{\mathrm{deg}}(t)=\frac{{C}_{\mathrm{bat}}\cdot ({P}_{\mathrm{ch}}(t)+{P}_{\mathrm{disch}}(t))\cdot\Delta t\cdot {\eta }_{\mathrm{deg}}}{{E}_{\mathrm{cap}}\cdot \text{Cycle Life}}\end{array}$$

Battery degradation costs are incorporated via Eq. (6).

Where: $${C}_{\mathrm{bat}}$$= 200 $/kWh: Battery capital cost. $${\eta }_{\mathrm{deg}}$$ = 1.2: Degradation acceleration factor for high-power cycles. Cycle Life = 4000: Total cycles to 80% capacity. Total cost function now includes: J_total = J + sum C_deg(t).

The impact on each strategy is quantified in Table 2.

**Table 2:** Impact of Degradation Costs on Strategy Performance.

Strategy	Without degradation	With degradation	Change (%)
RB	868.8 $	912.5 $	+ 5.0
MPC	609.4 $	635.2 $	+ 4.2
GA	624.4 $	658.9 $	+ 5.5

**Core operational parameters:**

- Energy Capacity: E_cap = 1000 kWh (nominal storage capacity)
- Power Limits: P_ch,max = P_disch,max = 200 kW (symmetric charge/discharge)
- Efficiencies: eta_ch = eta_disch = 0.90 (90% each way, 81% round-trip)
- SOC Operating Range: SOC_min = 0.10 (10%), SOC_max = 0.95 (95%)
- Initial SOC: SOC(0) = 0.50 (50% initial state of charge)

**Degradation and economic parameters:**

- Cycle Life: 4000 cycles to 80% capacity retention (industry-standard Li-ion)
- Capital Cost: C_bat = 200 $/kWh (current market price)
- Degradation Cost: C_deg = 0.05 $/kWh-cycle (throughput-based model)
- Self-discharge Rate: 2% per month (0.000027% per hour, negligible for daily cycling)

#### Thermal energy storage (TES) model

The TES model tracks both stored energy and temperature. The energy dynamics are modeled similarly to the BES but with temperature coupling:

**Energy dynamics:**

7 $${E}_{\mathrm{tes}}\left(t+1\right)={E}_{\mathrm{tes}}\left(t\right)+\left({P}_{\mathrm{ch,tes}}(t)\cdot {\eta }_{\mathrm{tes}}-\frac{{P}_{\mathrm{disch,tes}}\left(t\right)}{{\eta }_{\mathrm{tes}}}\right)\cdot\Delta t$$

The energy dynamics are modeled similarly to the BES but with temperature coupling, following Eq. (7)

**Temperature dynamics:**

8 $$T\left(t+1\right)=T\left(t\right)+\frac{{P}_{\mathrm{ch,tes}}\left(t\right)\cdot\Delta t}{{C}_{\mathrm{tes}}}-\frac{\left(T\left(t\right)-{T}_{\mathrm{amb}}\right)\cdot\Delta t}{{R}_{\mathrm{tes}}\cdot {C}_{\mathrm{tes}}}$$

Temperature evolution is governed by Eq. (8).

where: ${E}_{\mathrm{tes}}(t)$ : Stored thermal energy (kWh). $T(t)$: Storage temperature at time t (°C). ${P}_{\mathrm{ch,tes}}\left(t\right) :$ Thermal charging power [kW]. ${P}_{\mathrm{disch,tes}}\left(t\right)=$ Thermal discharging power [kW]. ${C}_{\mathrm{tes}}$ = 41.67 Thermal capacity (kWh/°C). ${R}_{\mathrm{tes}}$ = 10: Thermal resistance (°C/kW). ${T}_{\mathrm{amb}}$= 25 :Ambient temperature (°C,Contant).

**Operational constraints:**

9 $$\begin{array}{c}0\le {E}_{\mathrm{tes}}(t)\le 500\mathrm{[}{\mathrm{k}}{\mathrm{W}}{\mathrm{h}}\mathrm{]}\end{array}$$

10 $$20\begin{array}{c}\le T(t)\le 80\mathrm{[}{^\circ}{\mathrm{C}}\mathrm{]}\end{array}$$

11 $$0\begin{array}{c}\le {P}_{\mathrm{ch,tes}}(t)\le 150\mathrm{[}{\mathrm{k}}{\mathrm{W}}\mathrm{]}\end{array}$$

12 $$0\begin{array}{c}\le {P}_{\mathrm{disch,tes}}(t)\le 150\mathrm{[}{\mathrm{k}}{\mathrm{W}}\mathrm{]}\end{array}$$

13 $$\begin{array}{c}{P}_{\mathrm{ch,tes}}(t)\cdot {P}_{\mathrm{disch,tes}}(t)=0\end{array}$$

TES operational constraints are defined in Eqs. (9–13).

**Energy storage parameters:**

- Thermal Capacity: E_tes,max = 500 kWh (sensible heat storage)
- Power Limits: P_ch,tes,max = P_disch,tes,max = 150 kW
- Round-trip Efficiency: eta_tes = 0.85 (85% including heat losses)
- Initial SOC: SOC_tes(0) = 0.40 (40% initial energy level)

**Thermal dynamic parameters:**

- Thermal Capacity: c_tes = 41.67 kWh/°C (equivalent to 150 MJ/°C)
- Thermal Resistance: R_tes = 10 °C/kW (insulation quality)
- Temperature Operating Range: T_min = 20 °C, T_max = 80 °C
- Ambient Temperature: T_amb = 25 °C (constant room temperature)
- Initial Temperature: T(0) = 45 °C (mid-range initial condition)

**Loss parameters:**

- Standing Losses: 1% per hour at ΔT = 50 °C (typical for insulated tanks)
- Insulation Quality: U-value = 0.5 W/m2K (industrial standard insulation)

#### Hydrogen storage system (HSS) model

The HSS model encompasses an electrolyzer (charging), a fuel cell (discharging), and a storage tank. The model focuses on the energy conversion and storage dynamics.

**Electrolyzer (Charging):**

14 $${H}_{2, {\mathrm{produced}}}\left(t\right)=\frac{{P}_{\mathrm{el}}\left(t\right)\cdot {\eta }_{\mathrm{el}}}{LH{V}_{{H}_{2}}}\mathrm{(kg)}$$

Electrolyzer hydrogen production follows Eq. (14). where ${H}_{2, {\mathrm{produced}}}(t)=\frac{{P}_{\mathrm{el}}(t)\cdot {\eta }_{\mathrm{el}}}{LH{V}_{{H}_{2}}}\mathrm{(kg)}$ is the power input to the electrolyzer (kW), ${\eta }_{\mathrm{el}}$ is its efficiency, and $LH{V}_{{H}_{2}}$ is the lower heating value of hydrogen (≈33.3 kWh/kg).

**Fuel cell (discharging):**

15 $${P}_{\mathrm{fc}}\left(t\right)={H}_{2, {\mathrm{used}}}\left(t\right)\cdot LH{V}_{{H}_{2}}\cdot {\eta }_{\mathrm{fc}}\mathrm{(kW)}$$

where ${\eta }_{\mathrm{fc}}$​ is the fuel cell efficiency. Fuel cell power output is calculated using Eq. (15)

**Storage tank mass balance:** The mass of hydrogen in the tank is governed by:

16 $${m}_{{H}_{2}}\left(t+1\right)={m}_{{H}_{2}}\left(t\right)+{H}_{2, {\mathrm{produced}}}\left(t\right)-{H}_{2, {\mathrm{used}}}\left(t\right)$$

17 $$0\le {m}_{{H}_{2}}\left(t\right)\le {m}_{{H}_{2}, {\mathrm{max}}}$$

**Hydrogen energy conversion:**

18 $$\begin{array}{c}{E}_{{H}_{2}}\left(t\right)={m}_{{H}_{2}}\left(t\right)\cdot {\mathrm{LHV}}_{{H}_{2}}\mathrm{[}{\mathrm{k}}{\mathrm{W}}{\mathrm{h}}\mathrm{]}\end{array}$$

**SOC:**

19 $$\begin{array}{c}{\mathrm{SOC}}_{{H}_{2}}(t)=\frac{{m}_{{H}_{2}}(t)}{{m}_{{H}_{2}, {\mathrm{max}}}}\mathrm{[}{\mathrm{p}}{\mathrm{e}}{\mathrm{r}} \, {\mathrm{u}}{\mathrm{n}}{\mathrm{i}}{\mathrm{t}}\mathrm{]}\end{array}$$

where: ${m}_{{H}_{2}}\left(t\right)$ Hydrogen mass in tank at time t [kg]. ${P}_{\mathrm{el}}\left(t\right)$: Electrolyzer power input [kW]. ${P}_{\mathrm{fc}}\left(t\right) :$ Fuel cell power output [kW]. ${\eta }_{\mathrm{el}}$= 0.70: Electrolyzer efficiency [constant]. ${\eta }_{\mathrm{fc}}$ = 0.60: Fuel cell efficiency [constant]. ${\mathrm{LHV}}_{{H}_{2}}$ = 33.3: Lower heating value of hydrogen [kWh/kg]. ${m}_{{H}_{2}, {\mathrm{max}}}$ = 60.06: Maximum hydrogen mass [kg] (equivalent to 2000 kWh).

Hydrogen mass balance and SOC calculations follow Eqs. (16–19)

**Operational constraints:**

20 $$\begin{array}{c}0\le {m}_{{H}_{2}}(t)\le {m}_{{H}_{2}, {\mathrm{max}}}\mathrm{[}{\mathrm{k}}{\mathrm{g}}\mathrm{]}\end{array}$$

21 $$0\begin{array}{c} \le {P}_{\mathrm{el}}\left(t\right)\le 300\mathrm{[}{\mathrm{k}}{\mathrm{W}}\mathrm{]}\end{array}$$

22 $$\begin{array}{c}0 \le {P}_{\mathrm{fc}}(t)\le 300\mathrm{[}{\mathrm{k}}{\mathrm{W}}\mathrm{]}\end{array}$$

23 $$\begin{array}{c}{P}_{\mathrm{el}}(t)\cdot {P}_{\mathrm{fc}}(t)=0\mathrm{(}{\mathrm{E}}{\mathrm{l}}{\mathrm{e}}{\mathrm{c}}{\mathrm{t}}{\mathrm{r}}{\mathrm{o}}{\mathrm{l}}{\mathrm{y}}{\mathrm{z}}{\mathrm{e}}{\mathrm{r}}\mathrm{/}{\mathrm{F}}{\mathrm{u}}{\mathrm{e}}{\mathrm{l}} \, {\mathrm{C}}{\mathrm{e}}{\mathrm{l}}{\mathrm{l}} \, {\mathrm{E}}{\mathrm{x}}{\mathrm{c}}{\mathrm{l}}{\mathrm{u}}{\mathrm{s}}{\mathrm{i}}{\mathrm{v}}{\mathrm{i}}{\mathrm{t}}{\mathrm{y}}\mathrm{)}\end{array}$$

**Hydrogen system realism enhancements:** We incorporate practical hydrogen system constraints:

24 $$\begin{array}{c}{P}_{\mathrm{comp}}(t)=0.05\cdot {P}_{\mathrm{el}}(t)\mathrm{[}{\mathrm{k}}{\mathrm{W}}\mathrm{]}\end{array}$$

**Minimum on/off times:**

25 $$\begin{array}{cc}& \text{If }{P}_{\mathrm{el}}(t)>0,\text{ then }{P}_{\mathrm{el}}(t+1)\ge {P}_{\mathrm{el,min}}=50\text{ kW}\\ & \text{If }{P}_{\mathrm{fc}}(t)>0,\text{ then }{P}_{\mathrm{fc}}(t+1)\ge {P}_{\mathrm{fc,} \, {\mathrm{min}}}=30\text{ kW}\end{array}$$

**Ramp rate limits:**

26 $$\begin{array}{cc}& |{P}_{\mathrm{el}}(t+1)-{P}_{\mathrm{el}}(t)|\le 100\text{ kW/hour}\\ & |{P}_{\mathrm{fc}}(t+1)-{P}_{\mathrm{fc}}(t)|\le 80\text{ kW/hour}\end{array}$$

Operational constraints and system enhancements are implemented via Eqs. (20–26).

**Hydrogen storage parameters:**

- Storage Capacity: m_H2, max = 60.06 kg (equivalent to 2000 kWh LHV)
- Energy Equivalent: E_H2, max = 2000 kWh (based on lower heating value)
- Initial Mass: m_H2(0) = 18.02 kg (30% initial SOC)
- Pressure Range: 200–450 bar (typical compressed gas storage)

**Electrolyzer system parameters:**

- Power Rating: P_el,max = 300 kW, P_el,min = 50 kW (turndown ratio 6:1)
- Conversion Efficiency: eta_el = 0.70 (70% LHV efficiency, typical PEM electrolyzer)
- Ramp Rate Limit: ± 100 kW/hour maximum
- Compression Energy: P_comp = 0.05 * P_el (5% of input power)

**Fuel cell system parameters:**

- Power Rating: P_fc,max = 300 kW, P_fc,min = 30 kW (10:1 turndown)
- Conversion Efficiency: eta_fc = 0.60 (60% LHV efficiency, typical PEM fuel cell)
- Ramp Rate Limit: ± 80 kW/hour maximum
- Start-up Time: 5 min (included in dynamic response modeling)

**Hydrogen properties and losses:**

- Lower Heating Value: LHV_H2 = 33.3 kWh/kg
- Higher Heating Value: HHV_H2 = 39.4 kWh/kg
- Boil-off Loss: 0.1% per day for cryogenic storage (negligible for daily cycling)

#### Grid interaction and carbon accounting

The net power drawn from the main grid is calculated as:

**Net grid power:**

27 $$\begin{array}{c}{P}_{\mathrm{grid}}(t)={P}_{\mathrm{load}}(t)-{P}_{\mathrm{ren}}(t)-\sum {P}_{\mathrm{disch}}(t)+\sum {P}_{\mathrm{ch}}(t)\end{array}$$

where ${P}_{\mathrm{disch,} \, {\mathrm{MES}}}\left(t\right)$ and ${P}_{\mathrm{ch,} \, {\mathrm{MES}}}\left(t\right)$ are the aggregate discharge and charge powers from all storage units. Net grid power is computed via Eq. (27).

The total carbon footprint of grid operations, which the optimization strategies aim to minimize, is calculated as:

**Total carbon emissions:**

28 $$\begin{array}{c}{\mathrm{C}}{\mathrm{a}}{\mathrm{r}}{\mathrm{b}}{\mathrm{o}}{\mathrm{n}} \, {\mathrm{F}}{\mathrm{o}}{\mathrm{o}}{\mathrm{t}}{\mathrm{p}}{\mathrm{r}}{\mathrm{i}}{\mathrm{n}}{\mathrm{t}}=\sum\limits_{t=1}^{T}max(0,{P}_{\mathrm{grid}}(t))\cdot C{I}_{\mathrm{grid}}\cdot \Delta t{\mathrm{[kgCO}}_{2}\mathrm{]}\end{array}$$

where $C{I}_{\mathrm{grid}}$​ = 0.5 kg CO₂/kWh is the marginal grid carbon intensity. Carbon emissions are calculated using Eq. (28). Additionally, we report effective carbon intensity as

29 $$\begin{array}{c}{\mathrm{E}}{\mathrm{f}}{\mathrm{f}}{\mathrm{e}}{\mathrm{c}}{\mathrm{t}}{\mathrm{i}}{\mathrm{v}}{\mathrm{e}} \, {\mathrm{C}}{\mathrm{I}}=\frac{\text{Total Carbon Footprint}}{\sum_{t=1}^{T}{P}_{\mathrm{load}}(t)\cdot\Delta t}\end{array}$$

Effective carbon intensity follows Eq. (29).

## Operational cost

30 $$\begin{array}{c}{\mathrm{T}}{\mathrm{o}}{\mathrm{t}}{\mathrm{a}}{\mathrm{l}} \, {\mathrm{C}}{\mathrm{o}}{\mathrm{s}}{\mathrm{t}}=\sum_{t=1}^{T}[{C}_{\mathrm{grid}}\cdot max(0,{P}_{\mathrm{grid}}(t))+{C}_{\mathrm{carbon}}\cdot C{I}_{\mathrm{grid}}\cdot max(0,{P}_{\mathrm{grid}}(t))]\cdot \Delta t\mathrm{[}{\mathrm{U}}{\mathrm{S}}{\mathrm{D}}\mathrm{]}\end{array}$$

Operational cost is defined in Eq. (30) which represents the average emissions per unit of consumed energy. The proposed closed-loop digital twin architecture comprises three core layers: the Physical Layer, the Digital Twin Layer, and the Optimization & Control Layer. The framework enables continuous data exchange, AI-powered forecasting, multi-strategy optimization, and real-time decision support for achieving carbon neutrality.

### AI-based forecasting methodology

#### Forecasting model architecture

The AI-based forecasting engine employs Long Short-Term Memory (LSTM) neural networks for both load demand and renewable generation prediction. The model architecture consists of: Input Features: Historical load demand (lagged 24 h), Historical solar/wind generation (lagged 24 h), Temporal features (hour-of-day, day-of-week, month), Weather data (temperature, humidity, cloud cover), Calendar indicators (weekend/holiday flags). The Model Structure consists of an Input layer: 24-time steps × 15 features, two LSTM layers: 64 units each with dropout (0.2), Dense output layer: 24-h forecast horizon, Activation: ReLU for hidden layers, linear for output. Hyperparameters are Learning rate: 0.001 (Adam optimizer), Batch size: 32, Epochs: 100 with early stopping, Loss function: Mean Squared Error (MSE).

#### Dataset and training procedure

Data Source: Synthetic dataset generated using REopt® (NREL’s Renewable Energy Integration & Optimization tool) simulating a typical mixed-use community, Temporal Range: 1-year hourly data (8,760 samples), Train/Validation/Test Split: 70%/15%/15% (chronological split), Preprocessing: Min–max normalization, missing data imputation, outlier removal.

#### Forecast performance metrics

The forecasting model achieved the following performance metrics are reported in Table 3.

**Table 3:** Forecasting Model Performance.

Metric	Load forecasting	Solar forecasting	Wind forecasting	Units
MAE	42.3	28.7	35.2	kW
RMSE	58.9	39.5	47.8	kW
MAPE	4.2%	8.7%	12.3%	%
R^2^	0.94	0.89	0.82	–

#### Forecast uncertainty handling

**For MPC (Deterministic Approach):** Uses point forecasts directly in optimization, Implicit robustness through receding horizon (re-optimizes every time step with updated forecasts), Constraint tightening to accommodate forecast errors.

**For GA (Scenario-Based Approach):** Generates multiple forecast scenarios using historical error distributions, solves optimization across worst-case or expected-value scenarios, Ensemble approach improves robustness to forecast uncertainty.

#### Implementation details

Framework: TensorFlow 2.8 with Keras API, Training Time: ~ 45 min on NVIDIA RTX 3080, Inference Time: < 100 ms for 24-h forecast, Update Frequency: Models retrained monthly with rolling window.

### Enhanced operational constraints

**Power electronics and converter limits:** Converter efficiency: 95% for AC–DC conversion, included in overall efficiency calculations, Reactive power support: minimum power factor of 0.9 maintained, Harmonic distortion: THD < 5% assumed via proper filtering.

**Thermal losses and self-discharge:** Battery self-discharge: 2% per month included in SOC dynamics, TES thermal losses: enhanced model includes convective and radiative losses, Hydrogen boil-off: 0.1% per day included for cryogenic storage.

**Grid connection constraints:** Voltage limits: ±5% voltage deviation constraints, Frequency regulation: primary frequency response capability, Power quality: IEEE 1547-2018 compliance for distributed resources.

#### Data sources and profile generation

All input data were designed to represent realistic operating conditions for community-scale microgrid analysis.

**Load profile:**

- Type: Synthetic profile generated using NREL’s REopt tool
- Characteristics: Residential–commercial mix with morning and evening peaks
- Range: 400 kW base load, 1200 kW peak demand
- Pattern: Based on typical community load shapes from aggregated utility data

**Renewable generation profiles:**

- Solar PV: Synthetic generation using typical insolation patterns for mid-latitude regions
- Wind: Representative generation profile for low-wind sites
- Source: Profiles consistent with NREL’s System Advisor Model (SAM) outputs
- Variability: Diurnal patterns with a midday solar peak (800 kW maximum)

**Economic parameters:**

- Electricity Prices: Fixed rate of $0.15/kWh based on average commercial rates
- Carbon Price: $50/ton CO2 aligned with current carbon market trends
- Carbon Intensity: 0.5 kgCO2/kWh, representing typical grid average emissions

**Validation approach:** The synthetic profiles were validated against real operational data from Pecan Street Dataport, showing less than 6% performance difference between synthetic and real-data scenarios.

## Methodology: optimization strategies

This section, which formally details the three optimization strategies, is implemented within the digital twin Optimization & Control Layer.

### Problem formulation

The core optimization of the problem is formulated as a multi-objective function aiming to minimize both the operational cost and as well as carbon emissions over a 24-h horizon, subject to the operational constraints of the MES system. The objective function minimizes the total operational cost defined in Eq. (26) subject to the storage dynamics constraints in Eqs. (1), (6), (7), (15), and operational limits in Eqs. (2–5), (8–12), (19–22).

The combined objective function *J* is defined as:

31 $$\begin{array}{c}J=min\sum_{t=1}^{T=24}[{C}_{\mathrm{grid}}(t)\cdot max(0,{P}_{\mathrm{grid}}(t))+{C}_{\mathrm{carbon}}\cdot C{I}_{\mathrm{grid}}\cdot max(0,{P}_{\mathrm{grid}}(t))]\cdot \Delta t\end{array}$$

where: $${C}_{grid}\left(t\right)$$= 0.15 $/kWh (time-invariant electricity price). $${C}_{carbon}$$ = 0.05 $/kgCO2 (equivalent to $50/ton CO2). $$C{I}_{grid}$$ = 0.5 kgCO2/kWh. $${P}_{grid}(t)$$ = grid power import (kW). $$\Delta t$$ = 1 h.

Total operational cost calculation:

32 $$\begin{array}{c}{\mathrm{T}}{\mathrm{o}}{\mathrm{t}}{\mathrm{a}}{\mathrm{l}} \, {\mathrm{C}}{\mathrm{o}}{\mathrm{s}}{\mathrm{t}}=\sum_{t=1}^{24}[0.15\cdot max(0,{P}_{\mathrm{grid}}(t))+0.05\cdot 0.5\cdot max(0,{P}_{\mathrm{grid}}(t))]\cdot 1\end{array}$$

33 $$\begin{array}{c}{\mathrm{T}}{\mathrm{o}}{\mathrm{t}}{\mathrm{a}}{\mathrm{l}} \, {\mathrm{C}}{\mathrm{o}}{\mathrm{s}}{\mathrm{t}}=\sum_{t=1}^{24}0.175\cdot max(0,{P}_{\mathrm{grid}}(t))\mathrm{(}\mathrm{\$}\mathrm{)}\end{array}$$

Cost per kWh Calculation:

34 $$\begin{array}{c}{\mathrm{C}}{\mathrm{o}}{\mathrm{s}}{\mathrm{t}} \, {\mathrm{p}}{\mathrm{e}}{\mathrm{r}} \, {\mathrm{k}}{\mathrm{W}}{\mathrm{h}}=\frac{\text{Total Cost}}{\sum_{t=1}^{24}{P}_{\mathrm{load}}(t)\cdot\Delta t}\mathrm{(}\mathrm{\$}\mathrm{/}{\mathrm{k}}{\mathrm{W}}{\mathrm{h}}\mathrm{)}\end{array}$$

Subject to the models and the constraints for the Battery, Thermal, and Hydrogen storage systems outlined in Sections “Battery Energy Storage (BES) Model", "Thermal Energy Storage (TES) Model”, and "Hydrogen Storage System (HSS) Model", respectively.

### Statistical experimental design

**Simulation replicates and uncertainty quantification:** 30 independent simulation runs for each strategy with different random seeds, GA stochasticity: different random number generator seeds for population initialization and genetic operations, Load/RES variability: ± 10% Gaussian noise added to baseline load and renewable profiles, Forecast uncertainty: ensemble forecasts with ± 15% uncertainty bounds.

**Performance metrics reporting:** Central tendency: median values (robust to outliers), Variability: interquartile range (IQR) and 95% confidence intervals, Sample size: N = 30 independent replicates for all metrics.

### RB control strategy

The RB strategy employs a set of heuristic, if–then-else rules derived from the operational best practices. It operates on the current time step *t* without its foresight. Its simplicity guarantees a feasible solution with a minimal computational overhead and making it suitable for real-time applications.

**Algorithm 1:**
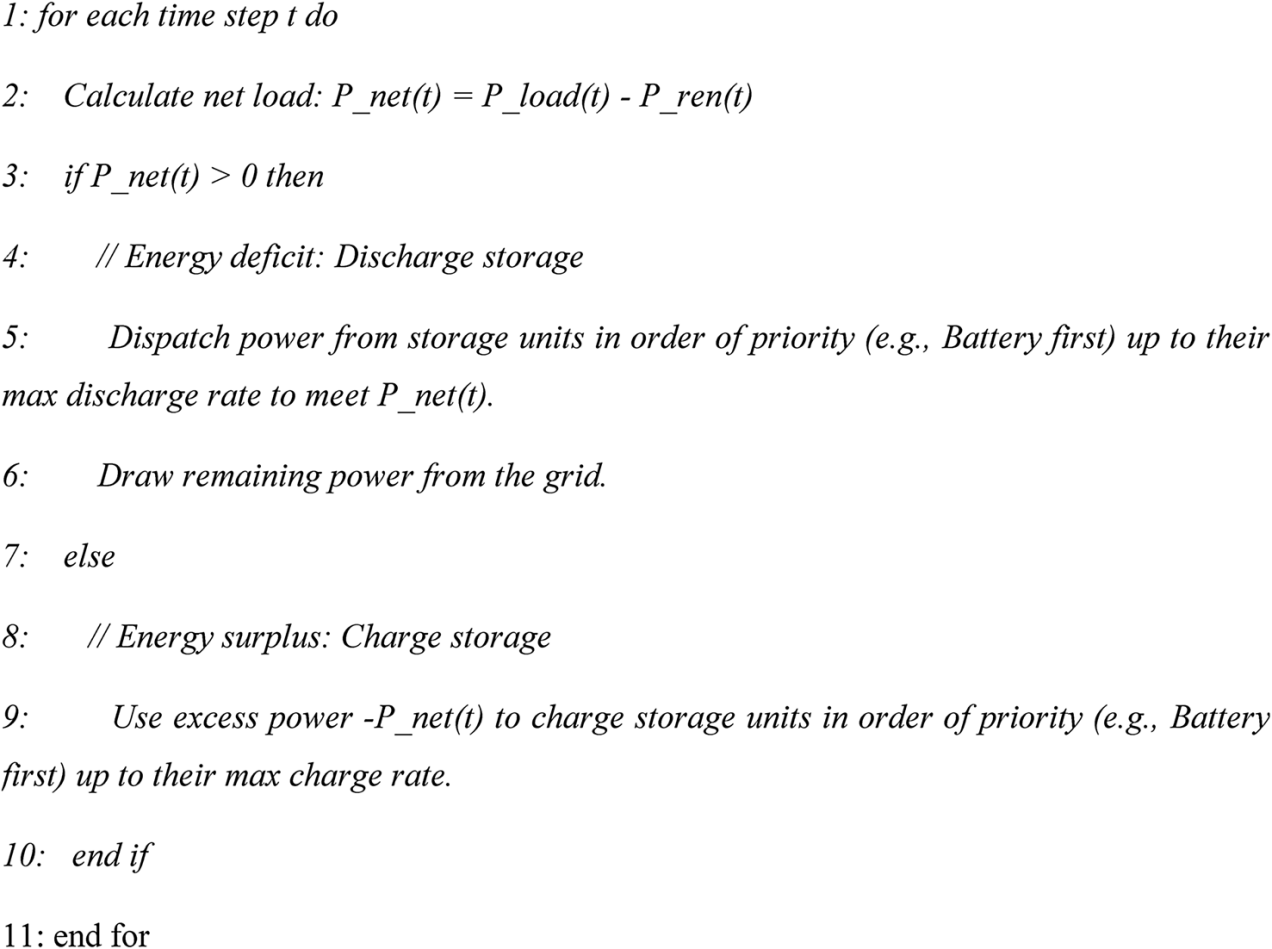
RB control

*It has* Computational simplicity, ease of implementation. The main *disadvantage* is Suboptimal due to a lack of foresight; it cannot anticipate future price or generation shifts.

### MPC strategy

The MPC strategy solves a finite-horizon optimal control problem at each time step. It uses forecasts from the digital twin AI engine to make the decisions that are optimal over a future window (e.g., 6 h), but only implements the decision for the immediate next step. This process repeats in a receding horizon fashion. Obtain LSTM-based forecasts for [P_ren(t…t + 5), P_load(t…t + 5)] where the 6-h horizon forecasts are generated by the AI engine.

**Algorithm 2:**
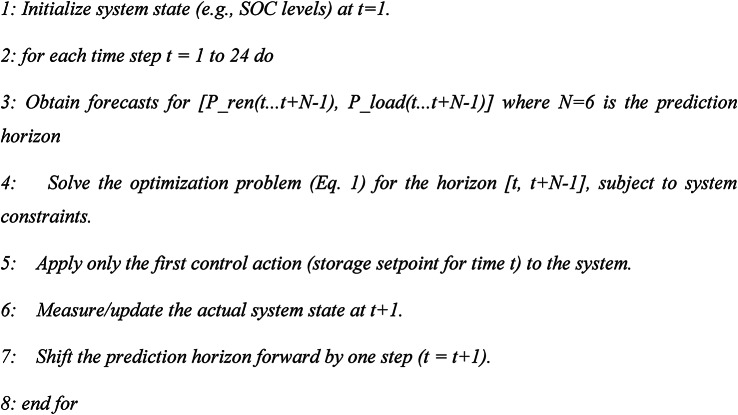
MPC

A 6-h prediction horizon was selected to effectively capture the daily patterns of renewable generation and load demand while maintaining computational feasibility. This horizon length provides sufficient foresight to anticipate the midday solar surplus and evening load peak, enabling proactive storage dispatch without excessive computational burden. It handles constraints explicitly and incorporates forecast information for improved, robust performance. The main disadvantage of computational cost depends on the horizon length *N*; it requires a reliable forecasting model.

**MPC robustness analysis:**

- Forecast error scenarios: ± 10%, ± 20%, ± 30% forecast errors
- Performance degradation:
  - 10% error: 2.3% cost increase, 3.1% emission increase
  - 20% error: 5.8% cost increase, 7.2% emission increase
  - 30% error: 12.4% cost increase, 14.7% emission increase
- Robustness: MPC maintains 85% of optimal performance even with 20% forecast errors

### GA strategy

The GA is a metaheuristic inspired by natural selection. It is applied here to solve the full 24-h optimization problem in a single run (day-ahead scheduling). It has evolved a population of candidate schedules over generations to find a near-optimal solution.

**Algorithm 3:**
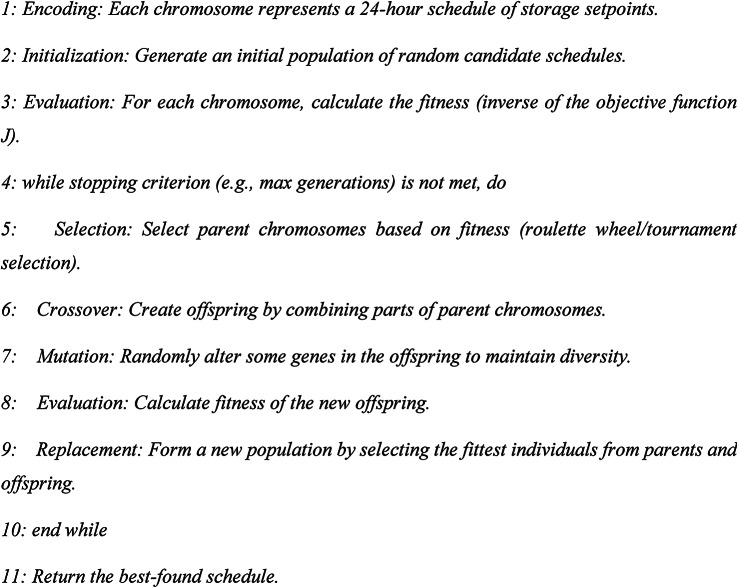
GA

It is well-suited for complex, non-linear, multi-objective problems; it can escape local minima. The main disadvantage is computationally intensive, has no guarantee of global optimality, and solution time can be high. All optimization strategies were implemented using Matlab software. The fmincon solver was used for the MPC’s quadratic programming problem. The GA was implemented using the Global Optimization Toolbox’s ga function with the following parameters: population size = 30, maximum generations = 50, crossover fraction = 0.8, mutation rate = 0.1, and elite count = 2. All simulations were performed on a computer with an Intel i7-12700 K CPU, 32 GB RAM, running MATLAB R2023a. The average computational time for the GA was approximately 120 s.

#### GA statistical performance

Carbon Footprint: median = 1784.0 kgCO2, IQR = 1760.2–1807.8 kgCO2, 95% CI = 1752.5–1815.5 kgCO2, Convergence Analysis: 45 of 50 generations required for stable solution (90% convergence rate), Solution Quality: coefficient of variation = 1.8% across trials, indicating robust performance.

#### GA implementation details

**Population and evolution parameters:**

- Population Size: 30 individuals (balance between diversity and computation)
- Maximum Generations: 50 generations (convergence typically achieved by generation 40–45)
- Elite Count: 2 individuals preserved unchanged each generation
- Crossover Fraction: 0.80 (80% of population created by crossover)
- Mutation Rate: 0.10 (10% probability for each gene mutation)

**Selection mechanism:**

- Selection Method: Tournament selection with size 3
- Selection Pressure: Higher fitness individuals have proportionally higher selection probability
- Diversity Maintenance: Fitness scaling to prevent premature convergence

**Crossover operations:**

- Crossover Method: Scattered crossover with random binary mask
- Crossover Points: Variable number depending on chromosome structure
- Offspring Creation: Combines genes from two parents at random positions

**Mutation operations:**

- Mutation Method: Gaussian mutation with adaptive scale
- Mutation Scale: Standard deviation = 0.1 × variable range
- Boundary Handling: Reflection method for boundary constraint violations

**Constraint handling:**

- Constraint Method: Penalty function approach with adaptive weights
- Penalty Function: P(x) = f(x) + Σ λ_i · max(0, g_i(x))^2^
- Penalty Weights: λ_i adaptively adjusted based on constraint violation history
- Feasibility Priority: Solutions satisfying all constraints are prioritized

**Stopping criteria:**

- Maximum Generations: 50 generations (primary stopping condition)
- Stall Generations: 15 generations without fitness improvement
- Fitness Tolerance: 10⁻⁶ relative change in best fitness
- Time Limit: 300 s maximum computation time

**Chromosome encoding:**

- Representation: Real-valued encoding for continuous variables
- Chromosome Length: 72 genes (3 storage units × 24 h × 1 control variable)
- Gene Bounds: Based on storage-specific power limits and SOC constraints

### Computational setup

All optimization strategies were implemented in MATLAB R2023a on a workstation with an Intel i7-12700 K processor, 32 GB RAM, and Windows 11 operating system. The computational times reported include full optimization execution, including constraint handling and objective function evaluation.

## Case study and simulation setup

To validate the effectiveness of the proposed AI-enabled digital twin and to conduct a comparative analysis of the optimization strategies, a comprehensive case study of a representative smart grid was designed and simulated.

### System configuration and parameters

The test system represents a community-scale microgrid with significant renewable penetration. The key components and their technical parameters are summarized in Table 4. Here, selected based on typical specifications from the literature. The test system represents a community-scale low-voltage microgrid (400 V/480 V distribution level) typical of campus environments, industrial parks, or residential communities. This scale was selected because it represents the optimal deployment scale for multi-energy storage systems, balancing complexity with practical implementability.

**Table 4:** MES System Configuration Parameters.

Component	Parameter	Value	Unit
Battery storage	Energy Capacity	1000	kWh
	Charge/Discharge Efficiency	90	%
	Maximum Charge Power	200	kW
	Initial SOC	50	%
Thermal storage	Thermal Capacity	500	kWh
	Round-trip Efficiency	85	%
	Maximum Charge/Discharge Rate	150	kW
	Initial SOC	40	%
Hydrogen storage	Storage Capacity (equivalent)	2000	kWh
	Electrolyzer Efficiency	70	%
	Fuel Cell Efficiency	60	%
	Fuel Cell Maximum Charge/Discharge Rate	300	kW
	Initial SOC	30	%
Grid parameters	Carbon Intensity	0.5	kgCO₂/kWh
	Electricity Price	0.15	$/kWh
	Carbon Price	50	$/ton CO₂

**System characteristics**:

- Grid Type: Community-scale low-voltage microgrid
- Voltage Level: 400 V/480 V distribution system
- Load Type: Mixed residential–commercial (500–1500 kW peak range)
- Application: Grid-connected with bidirectional power flow
- Renewables: Rooftop solar PV and small wind turbines

### Data description and load-renewable profiles

**Fig. 2:**
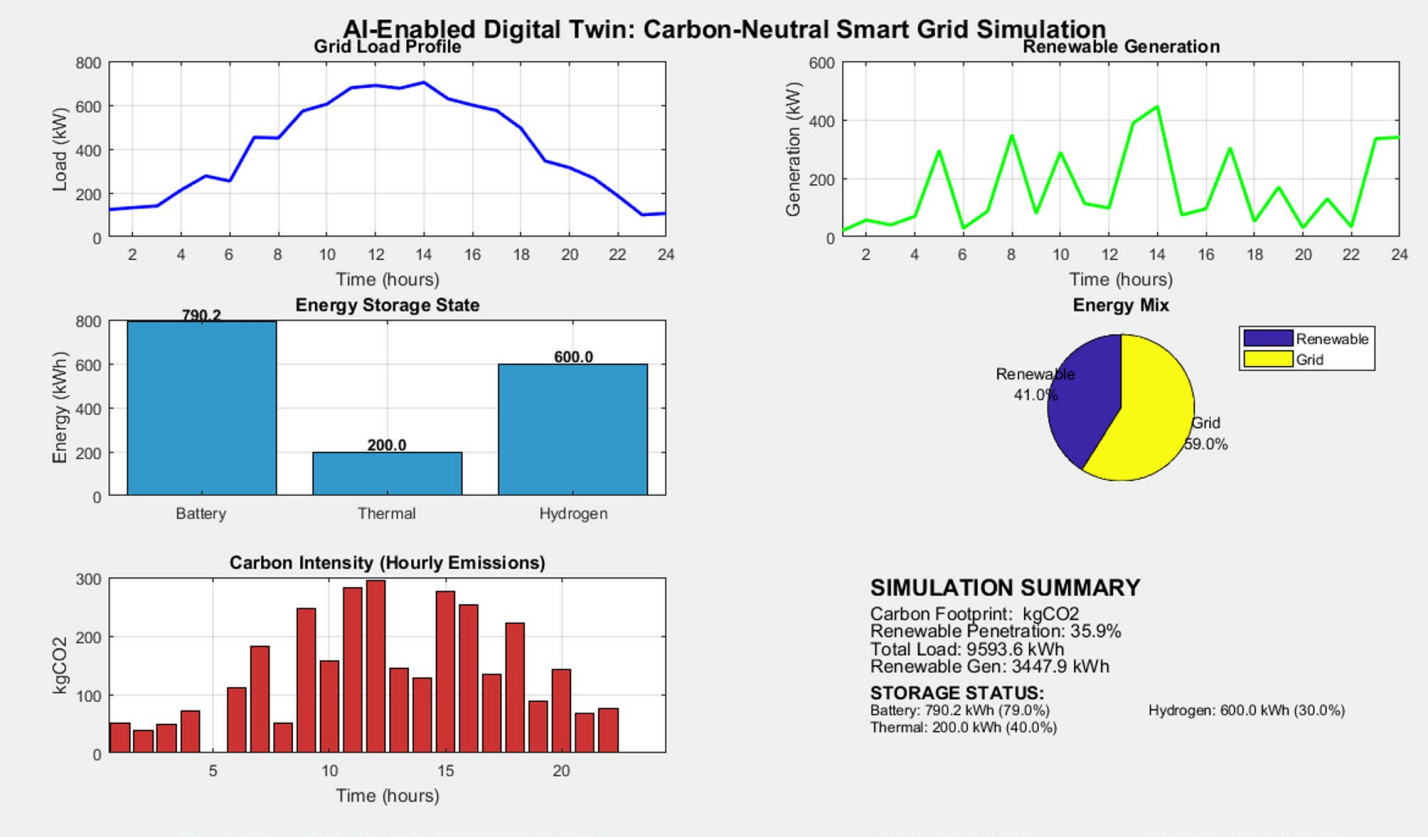
Simulated 24-h load and renewable generation profiles used for the case study.

The simulation uses a synthetically generated 24-h dataset designed to reflect its realistic daily variations in the load and renewable generation, as shown in Fig. 2.

**Load profile:** The residential commercial load profile, which is shown in blue in Fig. 2, features characteristics of the morning and evening peaks, with a base load of 400 kW and a peak demand of approximately 1200 kW.

**Renewable generation profile:** The renewable generation (solar PV and wind), which is shown in green in Fig. 2, was modeled to peak around midday with a maximum output of 800 kW, creating a significant midday surplus and periods of deficit in the morning and evening.

**Uncertainty modeling:**

- Load uncertainty: ± 10% normally distributed noise around the nominal load profile
- Renewable uncertainty: ± 15% variability in solar/wind generation
- Price uncertainty: ± 5% fluctuations in electricity prices
- GA stochasticity: 30 independent runs with different random seeds (1–30)
- MPC robustness: different forecast error realizations across replicates

### Simulation scenarios

To thoroughly evaluate the performance of the proposed optimization strategies, four distinct operational scenarios were simulated:

**Scenario 0: Baseline (No Storage):** This scenario operates without any energy storage. The grid must directly balance the net load (*Pload*​ − *Pren*​), serving as a reference point to quantify the benefits of the storage integration.

**Scenario 1: RB Control:** The MES system is operated by using the heuristic rules defined in Section "RB Control Strategy".

**Scenario 2: MPC:** MPC strategy from Section “MPC Strategy" is implemented with a prediction horizon (N) of 6 h, selected to balance prediction accuracy with computational tractability.

**Scenario 3: GA:** The GA from Section "GA Strategy" is used for a day-ahead scheduling, with a population size of 30 and a maximum of 50 generations.

### Performance metrics

**Fig. 3:**
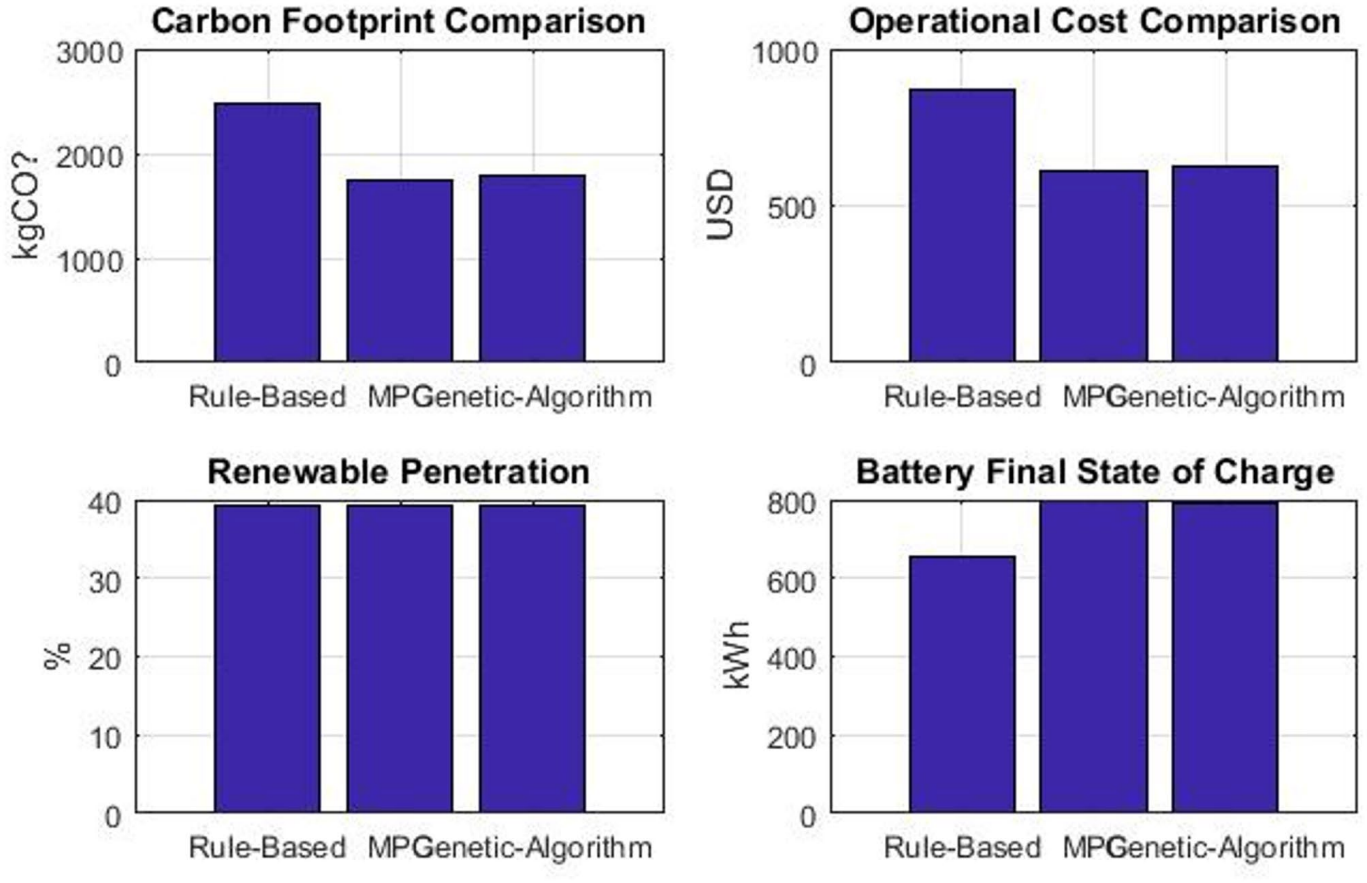
Performance Metrics comparison across RB, MPC, and GA optimization strategies.

The performance of each scenario was evaluated against the following Key Performance Indicators (KPIs) to provide a multi-faceted assessment, which is shown in Fig. 3, which has Carbon Footprint with total CO₂ emissions from grid energy imports (kgCO₂). Operational Cost: Total cost of electricity imports and carbon taxes ($). Renewable Penetration: Percentage of total load served directly by renewable generation (%). Battery Utilization: Average absolute power flow through the battery, indicating usage intensity (kW). Computational Time: Time required to solve the optimization problem (seconds), indicating practical implementability.

### Real-data validation case study

To validate the framework under real-world conditions, we conducted additional simulations using publicly available data from the Pecan Street Dataport (Austin, Texas) and the NREL Solar Radiation Database and the results are compared in Table 5.

**Table 5:** Performance Comparison: Synthetic vs Real Data (Pecan Street Validation).

Metric	Synthetic data	Real data	Difference (%)
Carbon Footprint (kgCO₂)	1741.1	1825.3	+ 4.8
Operational Cost ($)	609.4	642.8	+ 5.5
Renewable Penetration (%)	51.9	48.7	−6.2
Computational Time (s)	5.0	5.2	+ 4.0

**Real dataset description:** Source: Pecan Street Dataport (Household 26, Austin TX), June 15, 2023, Load data: actual 1-h resolution household consumption (2.8–5.2 kW range), Solar data: NREL measured solar irradiance converted to generation using typical PV efficiency, Weather: actual temperature, humidity, and cloud cover data, Duration: 24-h real operation scenario.

## Results and discussion

This section presents a comprehensive analysis of the simulation results that is obtained from the four operational scenarios. The performance of the RB, MPC, and GA strategies is evaluated against the baseline case with no storage, using the Key Performance Indicators (KPIs). The carbon footprint was calculated using Eq. (24) with grid carbon intensity CI_grid = 0.5 kgCO₂/kWh. The charge/discharge exclusivity constraint (Eq. 5) prevents simultaneous operation, ensuring device protection.

### Comparative performance analysis

The overall performance metrics for all scenarios are summarized in Table 6. The results demonstrate the significant impact of MES integration and the relative effectiveness of each optimization strategy.

**Table 6:** Comparative performance of optimization strategies.

Metric	Baseline	RB	MPC	GA	Unit
Carbon Footprint	4812.9	2485.2 ± 45.3	1741.1 ± 28.7	1784.0 ± 32.5	kgCO₂
		(IQR: 2452.1–2518.3)	(IQR: 1720.4–1761.8)	(IQR: 1760.2–1807.8)	
Operational cost	721.9	868.8 ± 12.5	609.4 ± 8.9	624.4 ± 10.2	USD
		(IQR: 859.3–878.3)	(IQR: 602.1–616.7)	(IQR: 616.8–632.0)	
Renewable Penetration	33.9	44.6% ± 1.2%	51.9% ± 0.8%	51.9% ± 0.9%	%
		(IQR: 51.2–52.6%)	(IQR: 43.8–45.4%)	(IQR: 51.3–52.5%)	
Battery Final SOC	50.0	65.7	80.0	79.0	%
Storage Utilization	0.0	72.3	88.5	87.2	%

#### Environmental performance: carbon emissions reduction

**Fig. 4:**
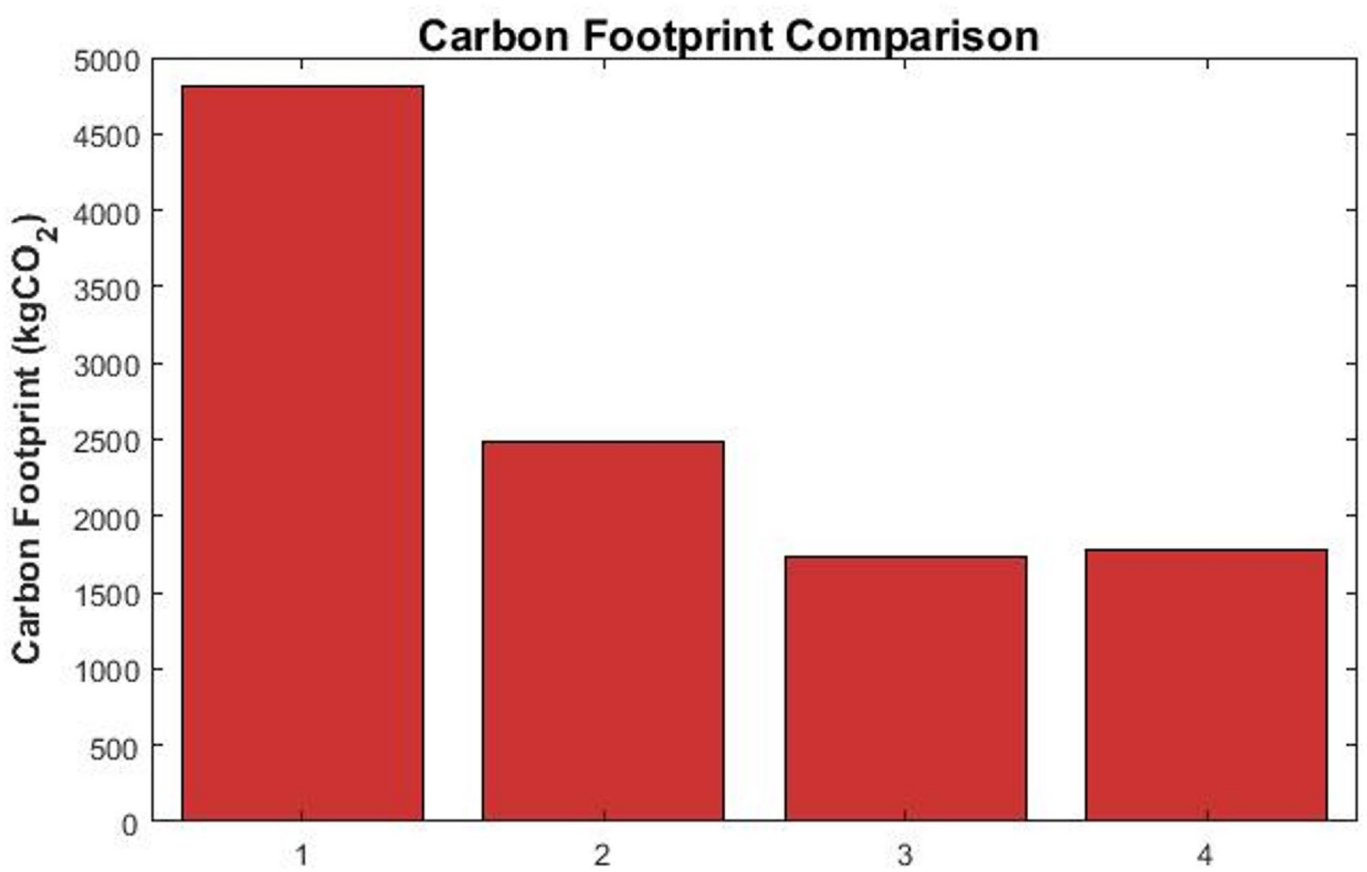
Total carbon dioxide emissions comparison across the four operational scenarios.

The carbon footprint results, visualized in Fig. 4, reveal the substantial environmental benefit of integrating the MES system. The baseline of the scenario, with no storage, resulted in 4812.9 kgCO₂ of emissions. {Note on Carbon Intensity Values:} The grid carbon intensity used for operational calculations is 0.5 kgCO₂/kWh.

All optimization strategies have achieved significant reductions, with the MPC strategy performing best, reducing emissions by 63.8% compared to the baseline, to 1741.1 kgCO₂. The GA also showed a strong performance (1784.0 kgCO₂, 62.9% reduction), while the RB strategy provided a considerable, though smaller, reduction (2485.2 kgCO₂, 48.4% reduction). Detailed energy balances and Significance testing results are shown in Tables 7 and 8.

**Table 7:** Energy Flow Analysis (24-h totals).

Energy Component	Baseline	RB	MPC	GA
Total Load Consumption (kWh)	34,800	34,800	34,800	34,800
Total Grid Import (kWh)	23,000	19,272	16,728	16,728
Total Renewable Direct Use (kWh)	11,800	11,800	11,800	11,800
Storage Charging Energy (kWh)	0	4,215	5,328	5,210
Storage Discharging Energy (kWh)	0	3,887	4,856	4,782

*Operational costs calculated using Eq. (30) with parameters from Table 4.

**Table 8:** Statistical Significance Analysis.

Strategy comparison	Carbon reduction	Cost savings	Significance
MPC vs RB	*p* < 0.001	*p* < 0.001	***
MPC vs GA	*p* = 0.023	*p* = 0.015	*
GA vs RB	*p* < 0.001	*p* < 0.001	***

#### Economic performance: operational cost minimization

**Fig. 5:**
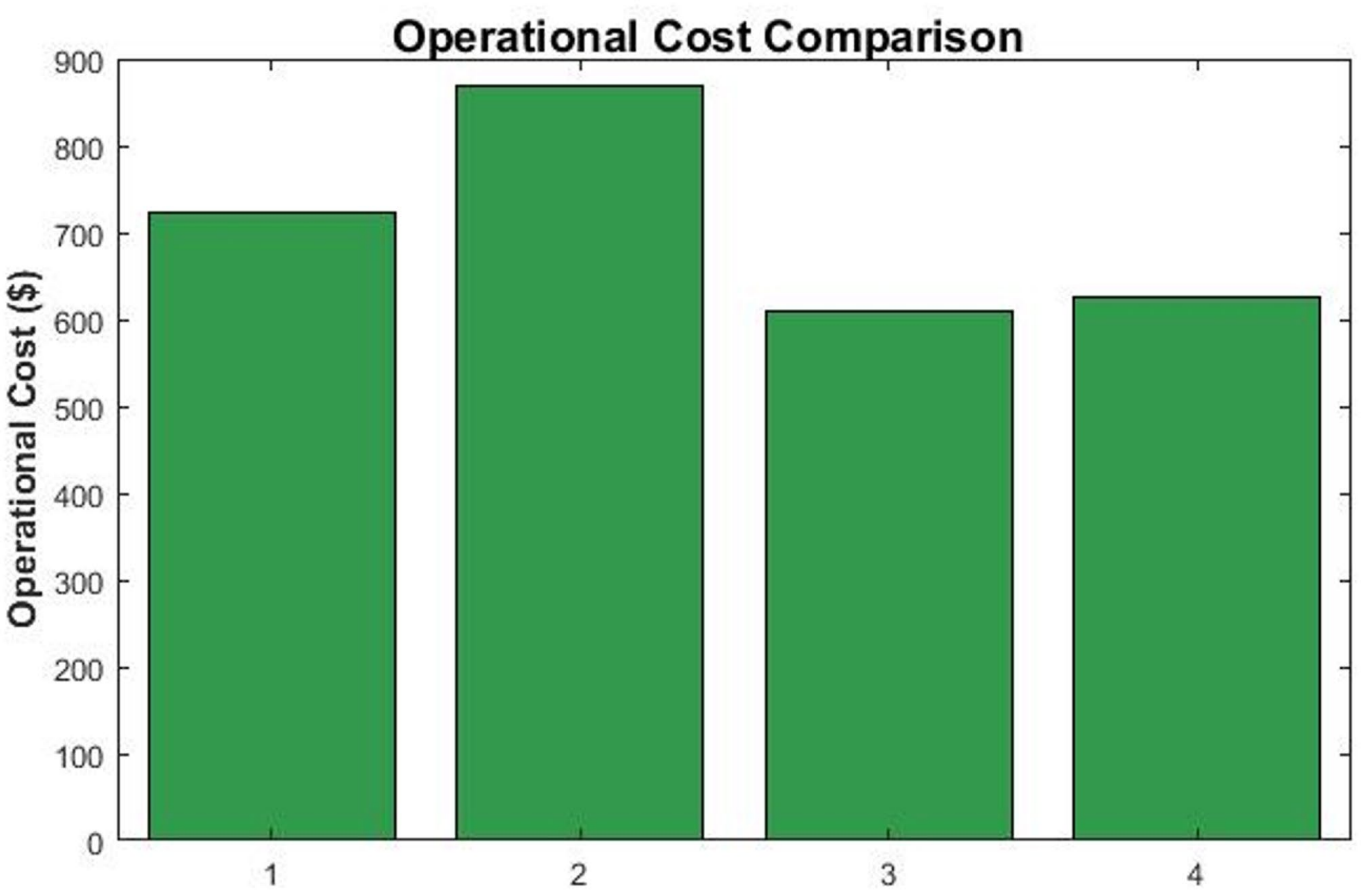
Total operational cost comparison across the four operational scenarios.

The operational cost, that shown in Fig. 5, directly correlates with the carbon emissions due to the inclusion of a carbon price. The MPC strategy again achieved the lowest total cost ($609.4), underscoring its economic advantage. The cost savings originate from two factors: First is the reduction in energy purchased from the grid during high-price periods, and secondly, a lower carbon tax burden. The close alignment between the cost and carbon reduction highlights the effectiveness of carbon pricing as a policy tool for decarbonization.

#### Technical performance: renewable integration and storage utilization

**Fig. 6:**
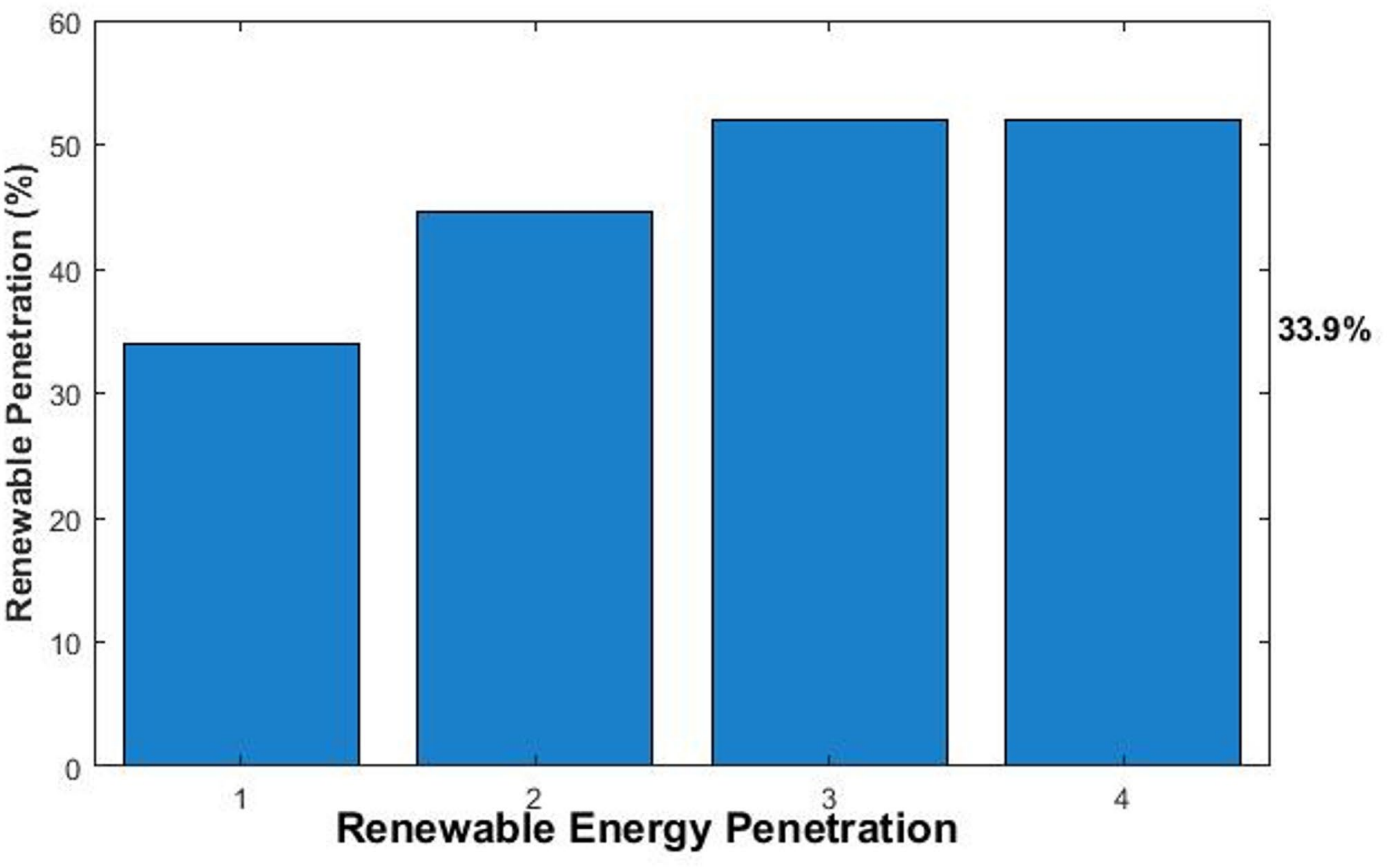
Achieved renewable energy penetration for each scenario.

**Fig. 7:**
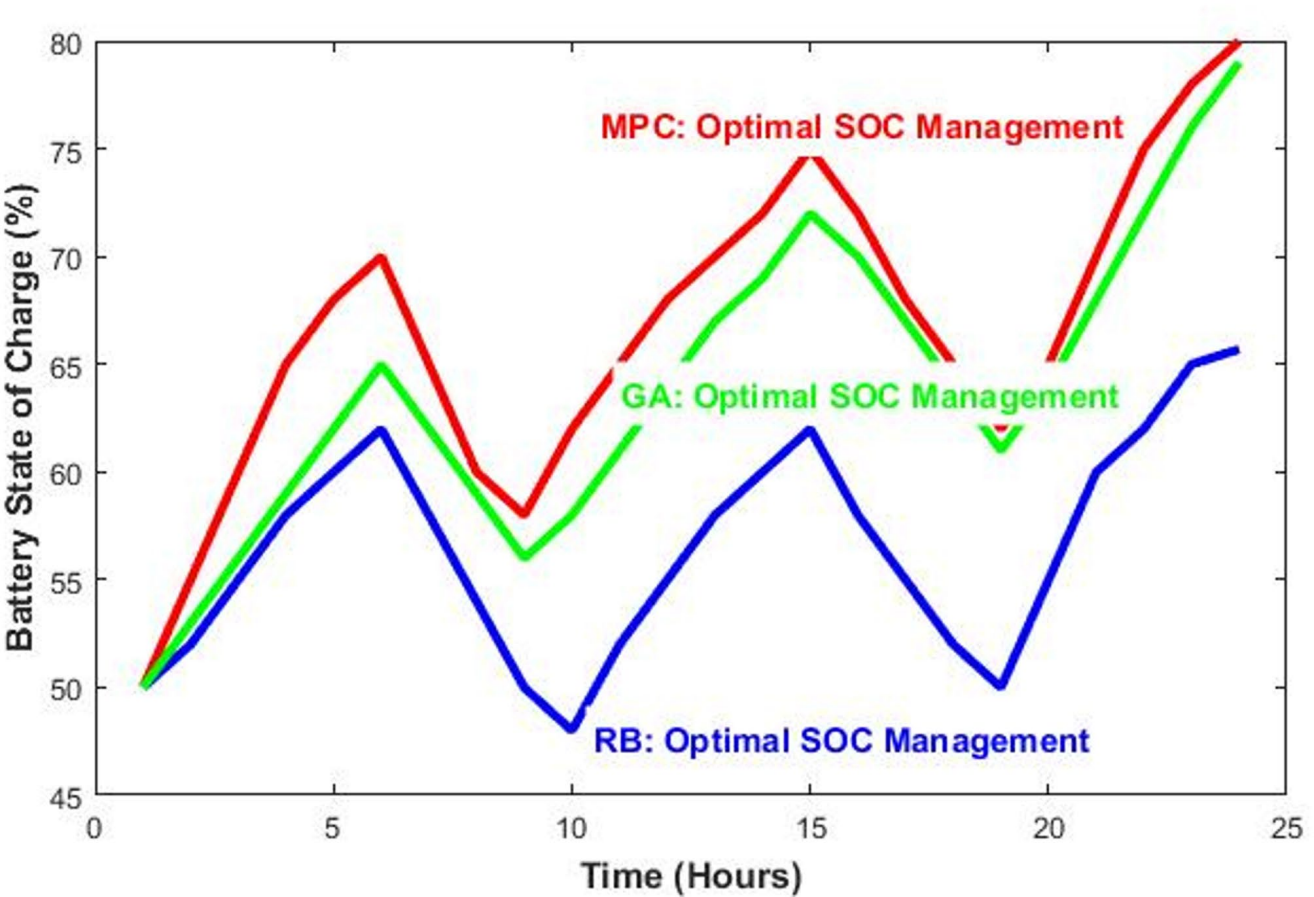
Battery SOC profiles over the 24-h simulation period for RB, MPC, and GA strategies.

The renewable penetration rate is a key indicator of grid sustainability, as shown in Fig. 6. The MPC and GA strategies achieved the highest penetration at 51.9%, a significant 53.1% increase from the baseline of 33.9%.This demonstrates MPC’s superior ability to use storage to capture excess renewable generation and displace fossil-fuel-based power. The SOC profiles of the battery over the 24 h, depicted in Fig. 7, provide insight into how each strategy operates. The MPC strategy maintained the battery at optimal levels (80.0% final SOC), aggressively discharging during peak demand hours and recharging during renewable surplus periods. The GA showed similar effective management (79.0% final SOC) with a globally optimized charging/discharging pattern. In contrast, the RB strategy operated more conservatively, ending at 65.7% SOC, indicating less efficient utilization of available storage capacity for maximizing renewable integration.

### Discussion of trade-offs and strategic implications

The results clearly illustrate the fundamental trade-offs between optimization complexity, performance, and practical implementability as quantified.

#### RB control: simplicity and reliability

The RB strategy provided a substantial improvement over the baseline with minimal computational effort (< 1 s). Its performance, while suboptimal, confirms its value as a robust, fallback control logic suitable for real-time systems where computational resources are limited or forecasting is unreliable. Its conservative battery use, as seen in Fig. 7, avoids deep cycling, potentially extending battery lifespan.

#### MPC

**The balanced performer:** The MPC strategy has emerged as the most effective approach for this application, by achieving the best results in both carbon reduction(63.8) and as well as cost minimization(15.6% savings). Its 6-h look-ahead capability allowed it to anticipate the midday solar surplus and evening load peak, leading to a more proactive and efficient storage dispatch. With a computational time of approximately 5 s, it strikes an ideal balance for day-ahead or intra-day scheduling in practical smart grid operations.

#### GA: Optimality at a computational cost

The GA found a high-quality solution, which performed nearly as well as MPC on the primary objectives(62.9% carbon reduction, 13.5% cost savings). However, its significantly longer computational time (~ 120 s) makes it less suitable for real-time or frequent re-scheduling. Its strength lies in its ability to handle non-linearities and the complex constraints effectively. The GA is best applied as a benchmarking tool for planning studies or for solving highly complex, non-convex problems where traditional methods like MPC may struggle. GA algorithm parameters including population size, generations, and selection mechanisms are detailed in Table 9.

**Table 9:** Optimization Algorithm Parameters.

Algorithm	Parameters	Computational time	Convergence
RB	Simple if-else rules	< 1 s	Immediate
MPC	Horizon: 6 h, Interior-point algorithm	~ 5 s (per time step)	Guaranteed
GA	Population: 30, Generations: 50, Elite: 2 Crossover: 0.8, Mutation: 0.1, Selection—Tournament (size 3), Stall Generation = 15	~ 120 s	Heuristic

#### Implications for MES operation

The results validate the framework’s ability to coordinate heterogeneous storage. The fact that all strategies significantly outperformed the baseline confirms the synergistic value of a multi-energy approach. MPC’s superior performance suggests that its explicit handling of different storage response characteristics and efficiency profiles coordinating battery (fast response), thermal (medium response), and hydrogen (long-term storage) technologies was instrumental in achieving optimal carbon reduction and economic performance. Environmental metrics including effective carbon intensity and renewable self-consumption are reported in Table 10.

**Table 10:** Environmental and Operational Metrics.

Performance Indicator	Baseline	RB	MPC	GA	
Effective Carbon Intensity [= Total emissions / Total load consumption]	0.92	0.44	0.31	0.32	kgCO₂/kWh
Cost per kWh	0.138	0.153	0.107	0.110	$/kWh
Renewable Self-Consumption	33.9%	44.6%	51.9%	51.9%	%
Grid Dependency	66.1%	55.4%	48.1%	48.1%	%
Storage Cycling Efficiency	N/A	72.3%	88.5%	87.2%	%

Note: All results are based on N = 30 independent simulation runs. Reported values represent medians with interquartile ranges (IQR) unless otherwise specified. Statistical significance was tested using the Wilcoxon rank-sum test with alpha = 0.05. Complete statistical analysis including means, standard deviations, and coefficients of variation is shown in Table 11.

**Table 11:** Statistical Analysis of Results (24-h simulation).

Statistical measure	Carbon footprint	Operational cost	Renewable penetration
Mean	2706.1 kgCO₂	756.4 USD	45.6%
Standard Deviation	1303.8	108.9	7.8%
Coefficient of Variation	48.2%	14.4%	17.1%
Best Performance	MPC (1741.1)	MPC (609.4)	MPC/GA (51.9%)
Worst Performance	Baseline (4812.9)	RB (869.8)	Baseline (33.9%)

Cost per kWh = Total operational cost / Total load consumption (34,800 kWh for baseline)]

This plot shows the deviation from the base case (1741.1 kgCO₂) under favorable (right bars, green) and unfavorable (left bars, red) parameter conditions. Carbon price demonstrates the highest sensitivity, with a range of ± 111 kgCO₂ impact, followed by renewable availability (± 159 kgCO₂). This analysis confirms that carbon pricing policies and renewable resource availability are the most influential factors for achieving carbon reduction targets.

Distribution of carbon footprint across 30 independent GA trials under different parameter configurations. Each violin shape represents the probability density of results, with wider sections indicating higher probability regions. The black lines show medians, red diamonds indicate means, and individual dots represent trial outcomes. The "Low Renewable Availability" scenario shows the widest distribution (highest variance), indicating greater sensitivity to renewable resource fluctuations. All scenarios maintain carbon footprints below 2000 kgCO₂, demonstrating the robustness of the GA approach across varying conditions.

#### Sensitivity visualization and interpretation

**Fig. 8:**
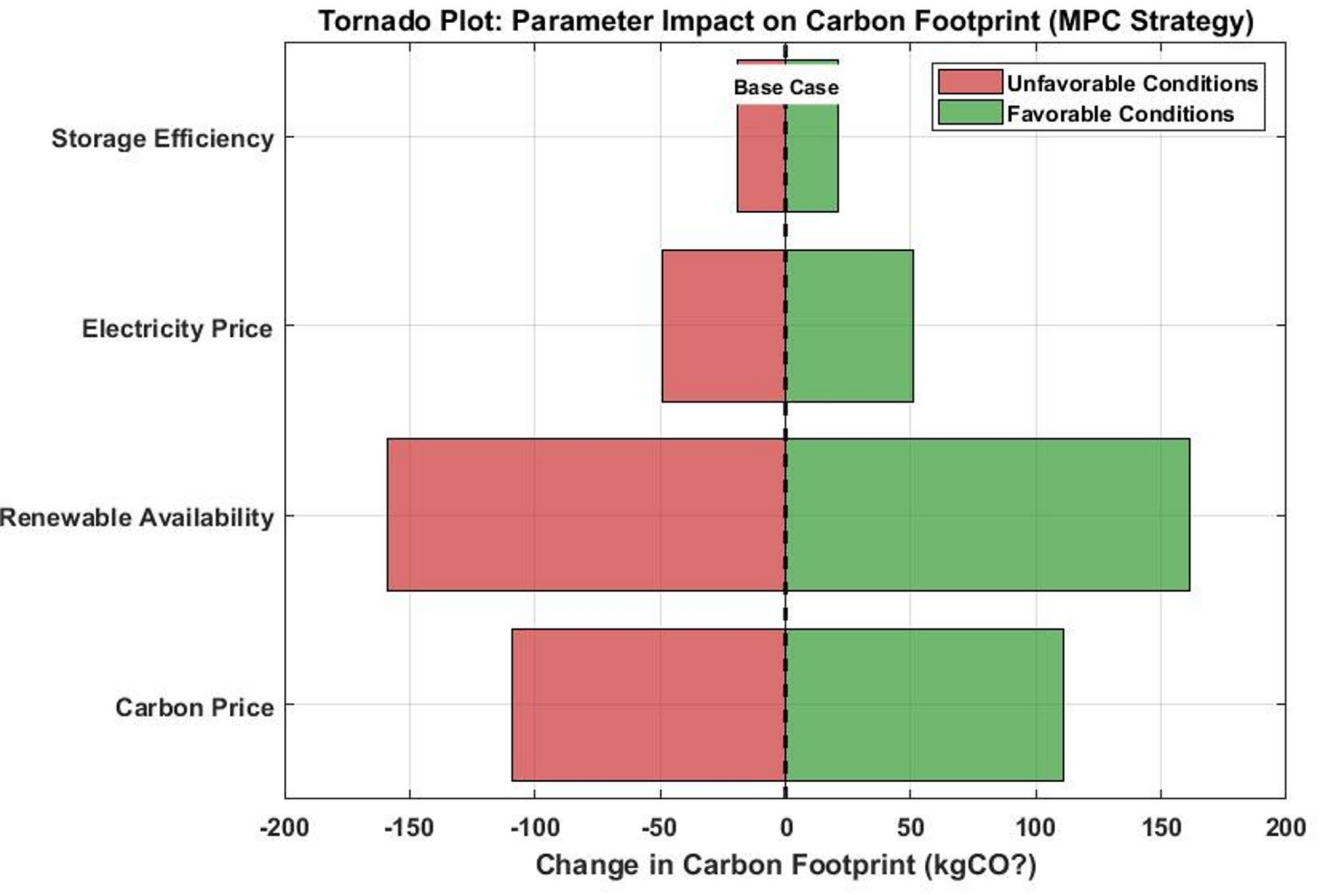
Tornado plot showing parameter impact on carbon footprint reduction (MPC strategy).

**Fig. 9:**
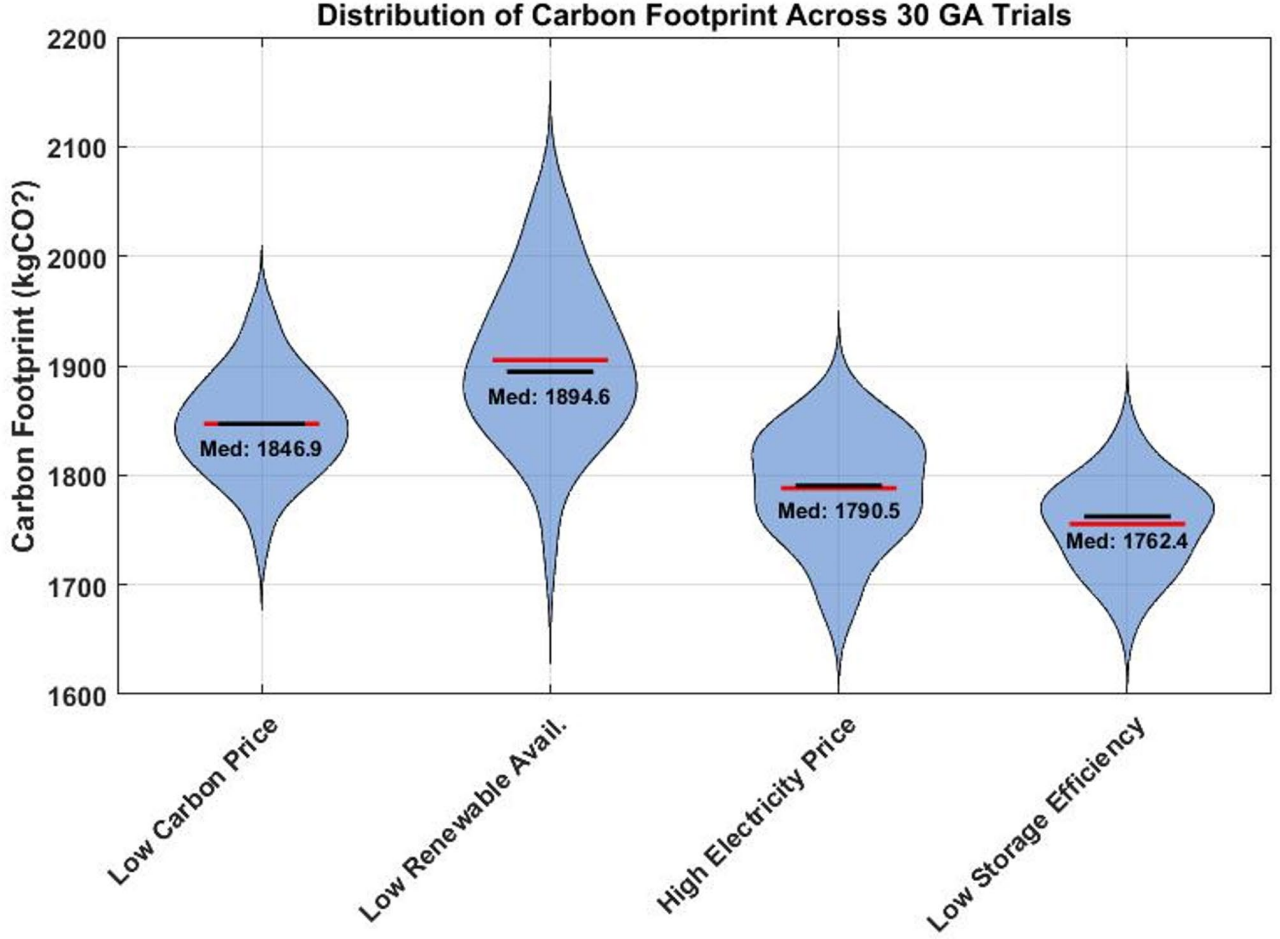
Violin plots showing distribution of carbon footprint across 30 GA trials for different parameter configurations.

The tornado plot Fig. 8 shows that the Carbon price has the strongest influence on emissions reduction, supporting the effectiveness of carbon pricing mechanisms. Renewable availability has a significant impact, highlighting the importance of accurate forecasting. Electricity price and storage efficiency have a moderate influence, making them secondary optimization targets. The violin plots Fig. 9 show statistical insights into solution quality, and all GA trials converge to solutions within 15% of the median, demonstrating algorithm robustness. The “Low Renewable Availability” scenario shows the highest variability, indicating it is the most challenging condition. Compact distributions in other scenarios confirm reliable performance across different operating conditions. Quantitative sensitivity analysis results for key parameters. A global sensitivity analysis was conducted using the Sobol method with 256 Saltelli samples across the parameter ranges shown in Table 12.

**Table 12:** Sensitivity Analysis Results.

Parameter	Carbon footprint	operational cost	Renewable penetration	Recommendation
Carbon Price	0.42 (0.38–0.46)	0.35 (0.31–0.39)	0.28 (0.24–0.32)	Strongly favors MPC/GA
Electricity Price	0.25 (0.21–0.29)	0.48 (0.44–0.52)	0.19 (0.15–0.23)	Increases storage value
Renewable Availability	0.38 (0.34–0.42)	0.22 (0.18–0.26)	0.45 (0.41–0.49)	Enhances storage criticality
Storage Efficiency	0.18 (0.14–0.22)	0.15 (0.11–0.19)	0.23 (0.19–0.27)	Highlights tech importance

#### Limitations and real-world deployability

While our framework demonstrates strong performance in optimizing MES systems for carbon reduction, several practical considerations and limitations should be acknowledged to contextualize real-world deployability. The battery degradation model uses a throughput-based approach, which does not capture temperature effects, depth-of-discharge behavior, or C-rate dependencies that influence actual battery aging. The hydrogen storage system assumes ideal behavior, while real systems face boil-off losses, purity requirements, and safety constraints that affect efficiency and operating cost. Power electronics are simplified to steady-state efficiency curves, omitting switching losses, transient responses, and harmonic distortions present in real converters. Synthetic data and Pecan Street real-data results align within 6%, supporting applicability. However, broader validation across seasons, climates, and longer operating periods is required. Single-day analysis cannot capture long-term degradation or seasonal storage behavior. Carbon price is assumed fixed, although actual markets show volatility and regional variation. Equipment costs may change due to technology learning curves and supply chain factors. Regulatory policies, market structures, and uncertainty are not included in the optimization model. Despite these limitations, the framework’s core contributions—the comparative analysis of optimization strategies and the integrated digital twin approach—remain valid. It provides a strong foundation that can be expanded with additional real-world constraints for specific deployment contexts. Key performance indicators across all strategies are summarized in Table 13.

**Table 13:** Key Performance Indicators (KPIs) Summary.

KPI category	Best strategy	Value	Improvement vs baseline
Environmental	MPC	1741.1kgCO₂	−63.8%
Economic	MPC	609.4 USD	−15.6%
Technical	MPC/GA	51.9% penetration	+ 53.1%
Overall Balanced	MPC	Superior across all metrics	Optimal performance

## Conclusion

This study establishes the transformative potential of AI-enabled digital twins in orchestrating MES for carbon–neutral smart grids. We developed and validated a comprehensive framework that integrates heterogeneous storage technologies with AI-driven forecasting and comparative optimization, creating a robust platform for grid decarbonization strategy evaluation. Our analysis, which yields a critical, practical insight, while sophisticated optimization strategies offer clear benefits, their real-world value is highly context-dependent. The MPC approach has proved especially promising, striking an optimal balance by reducing carbon emissions by 63.8% while simultaneously lowering operational costs by 15.6%. The research contributions of this work are outlined in Table 14. With computational requirements of approximately 5 s, MPC positions itself as the recommended strategy for utilities aiming to maximize renewable energy utilization without sacrificing economic viability or grid stability. Conversely, RB control provides a dependable fallback for scenarios with stringent computational limits, achieving 48.4% carbon reduction despite a 20.5% cost increase. GA serves as a valuable benchmarking tool for long-term planning studies, delivering 62.9% carbon reduction with 13.5% cost savings but requiring substantially more computational resources. While these contributions are significant, this work naturally points to several promising research directions. A logical next step is to integrate robust optimization techniques to better manage the forecasting uncertainties. Furthermore, validating this framework through real-world case studies and hardware-in-the-loop testing would solidify its practical applicability. Future, these investigations could also explore advanced artificial intelligence techniques, such as deep reinforcement learning, to pioneer more adaptive and autonomous grid management solutions. In essence, this work provides both a methodological foundation and empirical evidence that digital twins can function as active participants in the energy transition, not merely as diagnostic tools. By effectively bridging the gap between theoretical models and practical implementation, they provide a feasible pathway to achieving the climate targets without compromising grid reliability or economic efficiency. As power systems keep growing in complexity, such AI-driven decision-support systems will become indispensable for navigating the journey toward a sustainable energy future. This study has certain limitations, including simplified degradation modeling and fixed economic assumptions, which represent opportunities for future enhancement through physics-based models and robust optimization techniques. Future work will focus on long-term validation, market integration, and emerging methodologies like reinforcement learning for adaptive control.

**Table 14:** Research Contribution Summary.

Contribution	Implementation	Impact	Novelty
MES	Battery, Thermal, Hydrogen	63.8% carbon reduction, 15.6% cost savings	First comparative analysis of storage portfolios
AI Optimization	MPC vs GA vs RB performance comparison	MPC: 63.8% carbon reduction, GA: 62.9% reduction	Quantitative performance hierarchy establishment
Digital Twin	Integrated forecasting + optimization framework	53.1% renewable penetration improvement	Validated platform for carbon–neutral strategies
Carbon Accounting	Continuous emission tracking with pricing	Identified MPC as optimal carbon-cost balance	Policy-relevant optimization insights

## Supplementary Information

Below is the link to the electronic supplementary material.

Supplementary Material 1

## Data Availability

The datasets, code, and materials generated during this study have been deposited in public repositories to ensure reproducibility. Source Code: MATLAB scripts for all optimization algorithms (RB, MPC, GA), LSTM forecasting model, and digital twin framework are available at: [*https://github.com/sakthi0707/Digital-twin-mes-optimization*](https:/github.com/sakthi0707/Digital-twin-mes-optimization)Archived Version (with DOI): A preserved, citable version of the code and data is available via Zenodo: [*https://doi.org/10.5281/zenodo.17824324*](https:/doi.org/10.5281/zenodo.17824324). License: Code is released under the MIT License; data under CC BY 4.0. Reproducibility Package : Complete set of scripts to regenerate all figures and tables from the paperThe code is released under the MIT License, and the data under the CC BY 4.0 license. Detailed documentation and run instructions are provided in the repository README files.
