# Supplementary material for: A multi strategy optimization framework using AI digital twins for smart grid carbon emission reduction
Source: Sci Rep. 2026 Feb 12;16:8570. doi: 10.1038/s41598-026-38720-3 (PMC12976377; doi:10.1038/s41598-026-38720-3)
Supplement: Supplementary file 1 — Supplementary Material 1 [file 41598_2026_38720_MOESM1_ESM.docx]

**Supplementary Information**

**Appendix A: Results Reproducibility**

**A.1 Constants and Units**

**Table S.1:** System Constants and Parameters

| **Parameter** | **Value** | **Units** |
| --- | --- | --- |
| **Grid Parameters** |  |  |
| Grid Carbon Intensity (CI_grid) | 0.5 | kgCO₂/kWh |
| Electricity Price (C_grid) | 0.15 | $/kWh |
| Carbon Price (C_carbon) | 0.05 | $/kgCO₂ |
| Time Step (Δt) | 1 | hour |
| Simulation Duration | 24 | hours |
| **Battery Storage** |  |  |
| Capacity (E_cap) | 1000 | kWh |
| Max Charge/Discharge Rate | 200 | kW |
| Charging Efficiency (η_ch) | 0.90 | - |
| Discharging Efficiency (η_disch) | 0.90 | - |
| Initial SOC | 0.50 | p.u. |
| **Thermal Storage** |  |  |
| Capacity | 500 | kWh |
| Max Charge/Discharge Rate | 150 | kW |
| Efficiency (η_tes) | 0.85 | - |
| Initial SOC | 0.40 | p.u. |
| **Hydrogen Storage** |  |  |
| Capacity (equivalent) | 2000 | kWh |
| Electrolyzer Efficiency (η_el) | 0.70 | - |
| Fuel Cell Efficiency (η_fc) | 0.60 | - |
| Max Charge/Discharge Rate | 300 | kW |
| LHV of Hydrogen | 33.3 | kWh/kg |
| Initial SOC | 0.30 | p.u. |

**A.2 Objective Function**

**The multi-objective cost function is minimized over 24 hours:**

Total Cost J = sum from t=1 to 24 of [ C_grid × max(0, P_grid(t)) + C_carbon × CI_grid × max(0, P_grid(t)) ] × Δt

Where:

- P_grid(t) = P_load(t) - P_ren(t) - P_disch,MES(t) + P_ch,MES(t) [units: kW]
- C_grid = 0.15 [units: $/kWh]
- C_carbon = 0.05 [units: $/kgCO₂]
- CI_grid = 0.5 [units: kgCO₂/kWh]
- Δt = 1 [units: hour]

**A.3 Worked Example - Single Time Step (t=12)**

Assume at hour 12:

- P_load(12) = 1000 kW
- P_ren(12) = 600 kW
- P_disch,MES(12) = 200 kW (battery discharging)
- P_ch,MES(12) = 0 kW (no charging)

Step 1: Calculate Grid Power
P_grid(12) = 1000 - 600 - 200 + 0 = 200 kW

Step 2: Calculate Time Step Cost
Energy Cost = C_grid × max(0, P_grid(12)) × Δt = 0.15 × 200 × 1 = 30 $
Carbon Cost = C_carbon × CI_grid × max(0, P_grid(12)) × Δt = 0.05 × 0.5 × 200 × 1 = 5 $
Total Cost at t=12 = 30 + 5 = 35 $

Step 3: Calculate Carbon Emissions
Carbon Emissions at t=12 = CI_grid × max(0, P_grid(12)) × Δt = 0.5 × 200 × 1 = 100 kgCO₂

**A.4 Summary Numbers**

**Table S.2: Reconciliation of Reported Summary Numbers**

| Metric | Baseline | Rule-Based | MPC | GA | Units | Calculation Method |
| --- | --- | --- | --- | --- | --- | --- |
| **Carbon Footprint** | 4812.9 | 2485.2 | 1741.1 | 1784.0 | kgCO₂ | Σ[CI_grid × max(0,P_grid(t)) × Δt] |
| **Operational Cost** | 721.9 | 868.8 | 609.4 | 624.4 | $ | Σ[(C_grid + C_carbon×CI_grid) × max(0,P_grid(t)) × Δt] |
| **Renewable Penetration** | 33.9% | 44.6% | 51.9% | 51.9% | % | (Total renewable direct use / Total load) × 100 |
| **Effective Carbon Intensity** | 0.92 | 0.44 | 0.31 | 0.32 | kgCO₂/kWh | Total emissions / Total load consumption |
| **Cost per kWh** | 0.138 | 0.153 | 0.107 | 0.110 | $/kWh | Total cost / Total load consumption |

**Total Load Consumption:** 34,800 kWh (all scenarios)
**Total Renewable Generation:** 11,800 kWh (all scenarios)

**A.5 Dimensional Consistency Verification:**

All reported values maintain dimensional consistency:

- Energy calculations: [kW] × [hours] = [kWh]
- Carbon emissions: [kW] × [kgCO2/kWh] × [hours] = [kgCO2]
- Operational costs: [kW] × [$ / kWh] × [hours] = [USD]
- Efficiency calculations: unitless ratios (0–1) or percentages (0–100%)

Cross-verification confirms that all reported totals match dimensional analysis.

**Data Generation Methodology:**

Synthetic profiles were generated using the following approach:

- **Load Profiles**: Created using NREL's REopt tool with parameters for mixed-use communities, incorporating typical diurnal patterns, weekday–weekend variations, and seasonal characteristics.
- **Renewable Profiles**: Solar generation based on typical meteorological year data for mid-latitude locations with cloud cover variability. Wind profiles represent low-wind sites typical of community microgrid applications.
- **Economic Parameters**: Based on average 2023 utility rates and carbon market prices, held constant to isolate optimization strategy performance from price volatility effects.

All synthetic data maintain physical consistency (energy balances, power limits) and statistical realism compared to measured community microgrid data.

**A.6 Parameter Table**

**Table S.3 Complete Multi-Energy Storage System Parameter**

| **Parameter** | **Battery (BES)** | **Thermal (TES)** | **Hydrogen (HSS)** | **Unit** |
| --- | --- | --- | --- | --- |
| **Storage Capacity** | 1000 | 500 | 2000 | kWh |
| **Power Rating** | 200 | 150 | 300 | kW |
| **Min Power** | 0 | 0 | 50/30 | kW |
| **Round-trip Efficiency** | 81 | 85 | 42 | \% |
| **Component Efficiency** | 90/90 | 85 | 70/60 | \% |
| **Min SOC** | 10 | 0 | 5 | \% |
| **Max SOC** | 95 | 100 | 100 | \% |
| **Initial SOC** | 50 | 40 | 30 | \% |
| **Cycle Life** | 4000 | - | - | cycles |
| **Capital Cost** | 200 | 50 | 800 | \$/kWh |
| **Degradation Cost** | 0.05 | - | - | \$/kWh-cycle |
| **Self-discharge** | 2%/month | 1%/hour | 0.1%/day | - |
| **Ramp Rate** | Instant | Instant | 100/80 | kW/h |
| **Temperature Range** | - | 20-80 | - | °C |
| **Pressure Range** | - | - | 200-450 | bar |
